# Definition of Environmental Variables and Critical Periods to Evaluate Heat Tolerance in Large White Pigs Based on Single-Step Genomic Reaction Norms

**DOI:** 10.3389/fgene.2021.717409

**Published:** 2021-11-23

**Authors:** P. H. F. Freitas, J. S. Johnson, S. Chen, H. R. Oliveira, F. Tiezzi, S. F. Lázaro, Y. Huang, Y. Gu, A. P. Schinckel, L. F. Brito

**Affiliations:** ^1^ Department of Animal Sciences, Purdue University, West Lafayette, IN, United States; ^2^ USDA-ARS Livestock Behavior Research Unit, West Lafayette, IN, United States; ^3^ Farm Animal Genetic Resources Exploration and Innovation Key Laboratory of Sichuan Province, Sichuan Agricultural University, Chengdu, China; ^4^ Centre for Genetic Improvement of Livestock, Department of Animal Biosciences, University of Guelph, Guelph, ON, Canada; ^5^ Department of Animal Science, North Carolina State University, Raleigh, NC, United States; ^6^ Department of Animal Science, College of Agricultural and Veterinary Sciences, São Paulo State University (UNESP), Jaboticabal, Brazil; ^7^ Smithfield Premium Genetics, Rose Hill, NC, United States

**Keywords:** heat stress, heat susceptible, genotype-by-environment interaction, resilience, maternal-pig line

## Abstract

Properly quantifying environmental heat stress (HS) is still a major challenge in livestock breeding programs, especially as adverse climatic events become more common. The definition of critical periods and climatic variables to be used as the environmental gradient is a key step for genetically evaluating heat tolerance (HTol). Therefore, the main objectives of this study were to define the best critical periods and environmental variables (ENV) to evaluate HT and estimate variance components for HT in Large White pigs. The traits included in this study were ultrasound backfat thickness (BFT), ultrasound muscle depth (MDP), piglet weaning weight (WW), off-test weight (OTW), interval between farrowing (IBF), total number of piglets born (TNB), number of piglets born alive (NBA), number of piglets born dead (NBD), number of piglets weaned (WN), and weaning to estrus interval (IWE). Seven climatic variables based on public weather station data were compared based on three criteria, including the following: (1) strongest G×E estimate as measured by the slope term, (2) ENV yielding the highest theoretical accuracy of the genomic estimated breeding values (GEBV), and (3) variable yielding the highest distribution of GEBV per ENV. Relative humidity (for BFT, MDP, NBD, WN, and WW) and maximum temperature (for OTW, TNB, NBA, IBF, and IWE) are the recommended ENV based on the analyzed criteria. The acute HS (average of 30 days before the measurement date) is the critical period recommended for OTW, BFT, and MDP in the studied population. For WN, WW, IBF, and IWE, a period ranging from 34 days prior to farrowing up to weaning is recommended. For TNB, NBA, and NBD, the critical period from 20 days prior to breeding up to 30 days into gestation is recommended. The genetic correlation values indicate that the traits were largely (WN, WW, IBF, and IWE), moderately (OTW, TNB, and NBA), or weakly (MDP, BFT, and NBD) affected by G×E interactions. This study provides relevant recommendations of critical periods and climatic gradients for several traits in order to evaluate HS in Large White pigs. These observations demonstrate that HT in Large White pigs is heritable, and genetic progress can be achieved through genetic and genomic selection.

## Introduction

The incidence and severity of adverse climatic conditions is increasing as a result of climate change, and this has the potential to negatively impact livestock production (Vilas Boas Ribeiro et al., 2018; Bernabucci, 2019). Specifically, climate change-induced heat stress (HS) is considered a major welfare and production issue in the swine industry, especially as advances in genetic selection, nutrition, and management have increased pig performance (Kemp et al., 2018) and subsequently metabolic heat production (Stinn and Xin, 2014; Cabezón et al., 2016). Therefore, improving measures of swine performance and welfare under HS conditions is a primary objective of the global swine industry.

Improved climatic resilience can be achieved by crossbreeding thermally sensitive animals with more heat-tolerant (HT) breeds. Despite the favorable adaptation to HS, a result of these crosses is relatively low production (in comparison to the more productive breeds), which is not profitable under intensive production systems (Berman, 2011; Misztal, 2017). The losses in the swine industry associated with HS are mainly explained by reduced and inconsistent growth, decreased carcass quality, lower feed efficiency, poor sow performance, and increased mortality and morbidity (Renaudeau et al., 2012; Baumgard and Rhoads, 2013; Mayorga et al., 2018). Due to these negative effects and inherent differences in climate, nutrition, and management between nucleus and commercial swine farms, the consideration of genotype-by-environment (G×E) interaction is important for the identification of HT animals. Thereafter, HT animals can be used in reproduction schemes to improve the phenotypic performance of economically important traits (*i*.*e*., reproductive, growth, and body composition traits).

The genetic variability for HTol in livestock species has been previously reported (*e*.*g*., Carabaño et al., 2017; Misztal, 2017; Ansari-Mahyari et al., 2019; Zhang et al., 2019; Tiezzi et al., 2020). In general, the genetic analyses of HS in pigs are based on heat-load function for live or carcass weight in growing/finishing pigs and are usually emphasized in the sire lines (Zumbach et al., 2008; Fragomeni et al., 2016). Random regression models, more specifically linear reaction norm models (RNM), are commonly used to study G×E interactions (Rauw and Gomez-Raya, 2015), as they allow to better account for variations in HS (Fragomeni et al., 2016). With the use of RNM, the phenotypic values of each animal are randomly regressed on the environmental gradient. This approach generates a regression intercept and slope for each animal, allowing the study of across-environment genetic merit of individuals as well as their potential responsiveness to the environmental changes (Rauw and Gomez-Raya, 2015). Therefore, breeding values and genetic parameters can change gradually along the environmental gradient, and traits may have different means and (co)variances at different environments (Song et al., 2020). These models are applicable when there is a continuous environmental variable that can be used to explore the G×E interaction [*e*.*g*., contemporary group effect (CGe), temperature, relative humidity (RH), and temperature–humidity index (THI)].

A genetic or genomic evaluation that accounts for HS requires each record to be associated with some easy-to-measure environmental variables (ENV). Several studies have focused on alternative environmental gradients, such as estimated average performance of CGe (Li and Hermesch, 2016; Song et al., 2020; Chen et al., 2021) and climatic variables (Fragomeni et al., 2016; Tiezzi et al., 2020; Usala et al., 2021). However, the best environmental metrics and critical periods to be used when evaluating HTol in maternal-line pigs need to be comprehensively evaluated to generate accurate estimations of breeding values. In this regard, the main objectives of this study were to (1) define the best environmental descriptors based on public weather station information and critical periods to evaluate HTol and (2) estimate variance components for HTol for populations of Large White pigs (one of the main maternal-line breeds in the world).

## Materials and Methods

Animal welfare and ethics committee approval was not needed for this study, as all the datasets used were provided by commercial breeding operations.

### Phenotypic Records

All datasets were provided by the Smithfield Premium Genetics company (Rose Hill, North Carolina, US). Phenotypic nucleus-herd records were obtained from January 2004 to December 2019 for Large White animals distributed among 33 farms located across North America. The farm latitudes range from 34° to 42° north, and the longitudes range from -77° to -113°. Different management strategies have been used across farms, such as equipment to alleviate heat stress. However, these practices changed over time, and this information was not recorded. Moreover, the systematic effects included in the statistical models (*e*.*g*., contemporary group–CG) are assumed to account for these effects. The traits analyzed were related to body composition [ultrasound backfat thickness (BFT; mm) and ultrasound muscle depth (MDP; mm)], growth [weaning weight of the piglets (WW; kg) and off-test weight (OTW; kg; approximately measured at around 5.5 months of age)], and reproduction [interval between farrow (IBF; days), total number of piglets born (TNB), number of piglets born alive (NBA), number of piglets born dead (NBD), number of piglets weaned (WN), and weaning to estrus interval (IWE; days)]. Contemporary groups (CG) were defined by the concatenation of farrowing year, farrowing season, and farrowing farm for the reproductive traits and of birth year, birth season, and birth farm for the growth and body composition traits, respectively. The phenotypic datasets were edited independently for each trait by removing records deviating ±3.5 SD from the mean. In addition, CG with less than 10 records were removed from further analyses. The descriptive statistics and the number of animals for each studied trait are presented in Table 2.

### Pedigree and Genotypes

A total of 265,943 animals were included in the pedigree, which represented more than 10 generations. A total of 8,992 animals were genotyped using the PorcineSNP10K (8,652 SNPs for 886 animals), PorcineSNP50K (50,549 SNPs for 5,706 animals), PorcineSNP60K (57,019 SNPs for 865 animals), and PorcineSNP80K (64,577 SNPs for 1,535 animals) Bead Chips (Illumina, San Diego, CA, United States). In each SNP panel, animals with genotype call rate smaller than 0.90 were removed. Genotype imputation was performed using the FImpute v3 software (Sargolzaei et al., 2014). The missing genotypes were imputed first from the 10K to the 50K panel and then from the 50K or 60K to the 80K panel. The detailed imputation process was described in Chen *et al*. (2021). Quality control (QC) of genotype data consisted of removing SNPs with call rate below 0.90, minor allele frequency lower than 0.01, and difference between observed and expected heterozygous frequencies lower than 0.15. The genomic QC was implemented in the BLUPF90 family software (Misztal et al., 2018). In the end, 55,375 SNPs for 8,686 animals (7,017 female and 1,669 male) were included in the subsequent analyses.

### Weather Records, ENV Variables, and Critical Periods

Public weather station records for all farms were obtained from the Local Climatological Data at the National Oceanic and Atmosphere Administration (www.ncdc.noaa.gov/cdo-web/datatools/lcd?prior%2520=%2520N). Based on the location of the farms, climatic information was collected from the nearest airports. The average distance between an airport and the farm was 30 km (ranging from 7 to 64 km). Seven ENV were evaluated, including the average of mean temperature (MeanT), average of maximum temperature (MaxT), average of minimum temperature (MinT), dew point (DewP), average relative humidity (RH), average discomfort index (DI; Thom, 1959), and average THI calculated as in the Guide to Environmental Research on Animals (NRC, 1971). The average of the raw ENV was used as the environmental covariate. Supplementary Table S1 shows the description of the ENV range for TNB.

The critical periods evaluated in this study were chosen based on physiological knowledge (Johnson et al., 2015; Gonzalez-Rivas et al., 2020; Johnson et al., 2020) of heat stress and were trait dependent. The specific critical periods are shown in Table 1. Both chronic (*i*.*e*., HS during the entire grow-finish period; average of 120 days prior to measurement date) and acute (*i*.*e*., HS in the last 30 days prior to harvest) HS might have great impact in carcass-related traits, such as OTW, MDP, and BFT and, therefore, were evaluated for the mentioned traits. In addition, pigs exposed to *in utero* HS have been reported to develop a variety of postnatal phenotypes that prevent profitable production and compromised health and welfare in commercial production systems (Johnson et al., 2020; Maskal et al., 2020). These responses to HS are related to alteration of postnatal response, that is, change of core body temperature. With more intense HS, lactating sows have increased intestinal epithelial hyperpermeability and reduced reproduction (Mayorga et al., 2020). *In utero* heat stress can also impact long-term growth performance and decrease postnatal growth rates (Johnson et al., 2020; Maskal et al., 2020). Heat-stressed lactating sows also reduce feed intake as a mechanism to minimize metabolic heat production (Renaudeau et al., 2012; Williams et al., 2013; Cabezón et al., 2016). As such, in this study, two different critical periods were evaluated for IBF, IWE, WW, and WN: the first, a period ranging from the last stage of gestation (around day 80) until the farrowing date, and the second, a critical period ranging from the last stage of gestation throughout lactation until the weaning date. Regarding TNB, NBA, and NBD, only one critical period was evaluated. Early studies showed that the first weeks of gestation have a greater impact in the number of viable embryos (Edwards et al., 1968; Omtvedt et al., 1971; Wildt et al., 1975). Therefore, for TNB, NBA, and NBD, the average ENV ranging from 20 days before breeding to 30 days into gestation was considered.

**TABLE 1:** Description of the critical periods evaluated for each trait to be used for the genetic and genomic evaluation of heat stress in pigs.

Trait	Critical period
OTW	(1) 30 days prior to measurement date (2) 120 days prior to measurement date
MDP	
BFT	
WW	(1) 34 days prior to farrowing date (2) 34 days prior to farrowing date to weaning date
WN	
TNB	20 days prior to breeding to 30 days into gestation
NBA	
NBD	
IWE	34 days prior to farrowing to weaning date
IBF	

MDP, ultrasound muscle depth (mm); BFT, ultrasound backfat thickness (mm); WW, weaning weight (kg); OTW, off-test weight (kg); TNB, total number of piglets born; NBA, number of piglets born alive; NBD, number of piglets born dead; WN, number of piglets weaned; IWE, interval between wean to estrus (days); IBF, interval between farrows (days).

Chen *et al*. (2021), using data from the same population, performed an evaluation of G×E interaction based on CGe. In brief, they estimated variance components and individual breeding values for seven traits (OTW, MDP, BFT, TNB, NBA, WN, and WW), regressing their phenotype on the average effect of CG (Chen et al., 2021). The results obtained in the current study will be compared to the previous results based on the following comparison criteria: theoretical accuracy of genomic estimated breeding values (GEBV) and rank correlation between the GEBV of individuals regressed on the recommended ENV and CGe.

### Statistical Analyses

The best statistical model to describe each trait was defined in two steps: (1) the systematic (fixed) effects were defined based on the backwards elimination procedure using the *lm* function available in the software R (R Core Team, 2020) and (2) the random effects for each trait were defined using the BLUPF90 software (Masuda, 2018; Misztal et al., 2018) by comparing models, including a different combination of random effects [*i*.*e*., animal genetic effect, animal permanent environmental effect, and common environment (litter) effect]. Model comparisons were made, and the significant random effects were defined based on the Akaike Information Criterion (Akaike, 1973). Specific and detailed information regarding the definition of the models can be found in Chen *et al*. (2021). The final effects for each trait are shown in Table 2.

**TABLE 2:** Descriptive statistics of phenotypes and effects used for each trait.

Traits	Descriptive statistics				Effects	
	*N* of records	*N* of animals with records	SD	*N* of CG	Systematic	Random
OTW	101,541	101,541	25.63	256	Sex, BP, CG_G, WA (4)	*a*, *ce*
MDP	17,085	17,085	6.37	86	Sex, BP, CG_G, WA (4)	*a*, *ce*
BFT	17,086	17,086	4.1	87	Sex, BP, CG_G, WA (5)	*a*, *ce*
WW	24,280	24,280	1.88	74	Sex, BP, CG_G, WA (5)	*a*, *ce*
WN	6,059	2,665	2.79	97	FP, CG_R, FA (11)	*a*
TNB	172,984	71,151	3.41	476	FP, CG_R, FA (11)	*a*, *pe*, *ce*
NBA	172,418	71,016	3.2	476	FP, CG_R, FA (11)	*a*, *pe*, *ce*
NBD	171,062	69,586	0.37	422	FP, CG_R, FA (11)	*a*, *pe*, *ce*
IWE	128,675	52,544	4.52	450	FP, CG_R, FA (11)	*a*, *pe*, *ce*
IBF	104,917	45,780	10.08	411	FP, CG_R, FA (7)	*a*, *pe*

MDP, ultrasound muscle depth (mm); BFT, ultrasound backfat thickness (mm); WW, weaning weight (kg); OTW, off-test weight (kg); TNB, total number of piglets born; NBA, number of piglets born alive; NBD, number of piglets born dead; WN, number of piglets weaned; IWE, interval between wean to estrus (days); IBF, interval between farrows (days); *N*, number; SD, standard deviation; FP, farrowing parity; BP, birth parity; CG_R, reproduction contemporary group (defined by the concatenation of farrowing year, farrowing season, and farrowing farm); CG_G, growth contemporary group (defined by the concatenation of birth year, birth season, and birth farm); WA, weaning age divided in classes (number of classes inside parentheses); FA, farrowing age divided in classes (number of classes within parentheses); a , animal additive effect; pe , animal permanent environmental effect across parities; ce , litter effect.

After the definition of the significant effects to be included in the statistical model, RNM, using the single-step GBLUP approach, was implemented to obtain the reaction norm (RN) for each animal considering each ENV individually. Legendre orthogonal polynomials (order = 1; Kirkpatrick et al., 1990) were used to model the trajectory of phenotypic traits across environmental conditions. Variance components for all “trait × ENV” combinations were estimated using single-trait RNM and Bayesian inference, under a Markov chain Monte Carlo framework, using the THRGIBBS1F90 software (Misztal et al., 2002). Considering the corresponding effects for each trait (Table 2), the following model was used:

$$y_{\mathrm{ik}}=\alpha +\;x_{i}^{\prime }\beta +\omega {\hat{\varphi }}_{k}+\sum \left({\text{n}}_{0\text{i}}+{\text{n}}_{1\text{i}}{\hat{\varphi }}_{\text{k}}\right)\;+e_{\text{ik}},$$

where ${\text{y}}_{\text{i}}$ is the phenotypic observation of animal i, α is the intercept, $x_{i}^{'}$ is the row incidence vector for ββ , β is the vector of systematic effects described in Table 2, ω is the systematic regression coefficient of ${\text{y}}_{\text{i}}\;$ on the ENV, ${\hat{\text{φ}}}_{\text{k}}$ is the ENV vector (expressed as first-order Legendre polynomial) at the value *k*, ${\text{n}}_{0\text{i}}$ and ${\text{n}}_{1\text{i}}$ are the RN intercept and slope of animal i regressed on ${\hat{\text{φ}}}_{\text{k}}$ for the random effect n n∈{a,pe, ce} ( n∈{a,pe, ce} , a being the animal genetic effect, pe the animal permanent environmental effect, and ce the common environment effect, as described in Table 2 for each trait), and ${\text{e}}_{\text{ik}}$ is the random residual for the animal i. The assumptions regarding the random effects are as follows:

$$\left[\begin{array}{c} {\text{a}}_{0}\\ {\text{a}}_{1} \end{array}\right]∼\text{N}\left(0,\;H⊗\left[\begin{array}{cc} {\text{σ}}_{{\text{a}}_{0}}^{2} & {\text{σ}}_{{\text{a}}_{0}{\text{a}}_{1}}\\ {\text{σ}}_{{\text{a}}_{0}{\text{a}}_{1}} & {\text{σ}}_{{\text{a}}_{1}}^{2} \end{array}\right]\right),$$

and

$$\left[\begin{array}{c} \begin{array}{c} {\text{pe}}_{0}\\ {\text{pe}}_{1}\\ {\text{ce}}_{0} \end{array}\\ {\text{ce}}_{1}\\ \text{e} \end{array}\right]∼\text{N}\left(0,\;I⊗\left[\begin{array}{ccccc} {\text{σ}}_{{\text{pe}}_{0}}^{2} & {\text{σ}}_{{\text{pe}}_{0}{\text{pe}}_{1}} & 0 & 0 & 0\\ {\text{σ}}_{{\text{pe}}_{0}{\text{pe}}_{1}} & {\text{σ}}_{{\text{pe}}_{1}}^{2} & 0 & 0 & 0\\ 0 & 0 & {\text{σ}}_{{\text{ce}}_{1}}^{2} & {\text{σ}}_{{\text{ce}}_{0}{\text{ce}}_{1}} & 0\\ 0 & 0 & {\text{σ}}_{{\text{ce}}_{0}{\text{ce}}_{1}} & {\text{σ}}_{{\text{ce}}_{1}}^{2} & 0\\ 0 & 0 & 0 & 0 & {\text{σ}}_{\text{e}}^{2} \end{array}\right]\right),$$

where ${\text{σ}}_{{\text{n}}_{0}}^{2}$ , ${\text{σ}}_{{\text{n}}_{1}}^{2},$ and ${\text{σ}}_{{\text{n}}_{0}{\text{n}}_{1}}$ are the variance of coefficient ${\text{ n}}_{0\text{i}}$ , variance of coefficient ${\text{n}}_{1\text{i}}$ , and covariance between ${\text{n}}_{0\text{i}}$ and ${\text{n}}_{1\text{i}}$ , respectively, where n represents the random effects described above (*i*.*e*., a, pe, and ce), *e* is the residual variance, **A** is the pedigree-based relationship matrix, and **I** is an identity matrix. A chain containing a total of 600,000 iterations, with thin and burn-in of 60 and 300,000, respectively, was used for all traits except IBF. For IBF, a chain containing 900,000 iterations with thin and burn-in of 60 and 600,000 was used. These parameters allowed the model convergence for all traits analyzed in this study. The convergence was verified based on both graphical analyses and Raftery and Lewis criterion (Raftery and Lewis, 1992), both available in the Bayesian Output Analysis (Smith, 2007) package of the R software (R Core Team, 2020).

The same single-trait RNM previously mentioned in this study was used to perform ssGBLUP analyses by replacing the **A** by the **H** matrix (Misztal et al., 2009; Aguilar et al., 2010; Christensen and Lund, 2010). As the direct estimation of **H** is computationally demanding, **H** ^**−1**^ was calculated directly as in Aguilar *et al*. (2010):

$$H^{-1}=A^{-1}+\left[\begin{array}{cc} 0 & 0\\ 0 & \text{τ}{\left(\text{α}G-\text{β}A_{22}\right)}^{-1}-\text{ω}A_{22}^{-1} \end{array}\right],$$

where $A^{-1}$ is the inverse of the numerator relationship matrix A , $A_{22}^{-1}$ is the inverse of the subset of A related to the genotyped animals, and $G^{-1}$ is the inverse of the genomic relationship matrix G [calculated using the first method proposed by VanRaden (2008)]. For the construction of **H**, the default parameters from the BLUPF90 software (Masuda, 2018; Misztal et al., 2018) were used (*i*.*e*., *τ* = 1, *ω* = 1, *α* = 0.95, and *β* = 0.05). The parameters used for the comparison of the results are described in Section 2.3 and Section 2.5.

### Environmental Variable Selection

Three criteria were used to select the optimal ENV to analyze HTol based on the studied traits: (1) the ENV yielding the strongest G×E estimate as measured by the parameter ${\text{σ}}_{{\text{a}}_{1}}^{2}{\text{σ}}_{{\text{a}}_{1}}^{2}$ , (2) the ENV yielding the highest accuracy of GEBV for the slope, and (3) the ENV yielding the highest deviation of GEBV per ENV (*i*.*e*., allowing to more easily differentiate tolerant and susceptible individuals). Weights of 0.5, 0.3, and 0.2 were given to each criterion (1, 2, and 3, respectively) to facilitate the ENV selection. The climatic variable resulting in the highest final value was selected as the best indicator to evaluate HTol in Large White pigs.

### Heritability Estimates and Accuracy of GEBV

After obtaining the RN components, a genetic (co)variance matrix $\Gamma_{n}$ among values of ENV was calculated for each *n* random effect (described above) as in Tiezzi et al. (2020):

$$\Gamma_{n}=\;ФG_{n}{Ф}^{\prime },$$

where $G_{n}$ is the estimated (co)variance matrix between the intercept and slope terms for the corresponding *n* effect and Ф is a matrix of the number of rows equal to the number of unique values of the ENV and two columns (a vector of “1” and the standardized ENV). The heritability of a trait at each single value *k* of ENV (*h* ^2^_k_) was calculated as follows:

$${\text{h}}_{\text{k}}^{2}=\;\frac{\Gamma_{\mathrm{akk}}}{\text{Σ}\left(\Gamma_{\mathrm{nkk}}\right)+{\text{σ}}_{\text{e}}^{2}},$$

where ${\text{Γ}}_{\text{akk}}$ is the additive genetic variance for the ENV *k* and $\Gamma_{\mathrm{nkk}}$ is the variance for the *n* effects (*i*.*e*., a, pe, and ce) for the ENV k.

The theoretical accuracy of GEBV predicted for the slope and intercept of the trait in consideration of the animal i was calculated as follows:

$${\text{Acc}}_{\text{it}}=\sqrt{1-\frac{{\hat{\text{SD}}}_{\text{it}}^{2}}{\left(1+{\text{F}}_{\text{i}}\right){\hat{\text{σ}}}_{\text{at}}^{2}}},$$

where $\hat{{\text{SD}}_{\text{i}}}$ is the posterior standard deviation of GEBV for animal i for the RN of  t , being t the intercept or slope terms, ${\text{F}}_{\text{i}}$ is the inbreeding coefficient, and ${\hat{\sigma }}_{a}^{2}$ is the estimated variance of animal additive effect for the intercept or slope (Aguilar et al., 2020).

### Genetic Correlations Among Environments and Approximated Weighted Genetic Correlations Between Traits

Genotype-by-environment interaction can be confirmed, among other factors, by a genetic correlation lower than 0.70 across the range of ENV (Mulder and Bijma, 2007). The following equation was used to calculate the genetic correlation between the range of ENV:

$${\text{r}}_{\text{kk}\text{′}}=\frac{{\hat{\text{σ}}}_{{\text{u}}_{\text{kk}\text{′}}}}{\sqrt{{\hat{\text{σ}}}_{{\text{u}}_{\text{k}}}^{2}{\hat{\text{σ}}}_{{\text{u}}_{\text{k}\text{′}}}^{2}}},$$

where the covariance of additive genetic effects between ENV k and k′ is ${\hat{\text{σ}}}_{{\text{u}}_{\text{kk}\text{′}}}={\hat{\text{σ}}}_{{\text{a}}_{0}}^{2}+{\hat{\text{σ}}}_{{\text{a}}_{0}{\text{a}}_{1}}{\text{φ}}_{\text{k}}+{\hat{\text{σ}}}_{{\text{a}}_{0}{\text{a}}_{1}}{\text{φ}}_{\text{k}\text{′}}+{\hat{\text{σ}}}_{{\text{a}}_{1}}^{2}{\text{φ}}_{\text{k}}{\text{φ}}_{\text{k}\text{′}}$ .

The approximated weighted genetic correlation between the studied traits was assessed based on the correlation between the GEBV. Animals with a GEBV theoretical accuracy for the intercept and slope terms lower than 0.30 were removed from the calculations. The following approach was used (Almeida-de-Macedo et al., 2013):

$${\hat{\text{r}}}_{\text{g}\left(\text{xy}\right)}=\frac{\sum {\text{w}}_{\text{i}}\left({\text{x}}_{\text{i}}-\bar{\text{x}}\right)\left({\text{y}}_{\text{i}}-\bar{\text{y}}\right)/\sum {\text{w}}_{\text{i}}}{\sqrt{\frac{\sum {\text{w}}_{\text{i}}{\left({\text{x}}_{\text{i}}-\bar{\text{x}}\right)}^{2}}{\sum {\text{w}}_{\text{i}}}\times \frac{\sum {\text{w}}_{\text{i}}{\left({\text{y}}_{\text{i}}-\bar{\text{y}}\right)}^{2}}{\sum {\text{w}}_{\text{i}}}}},$$

where $\bar{\text{x}}=\frac{\sum {\text{w}}_{\text{i}}{\text{x}}_{\text{i}}}{\sum {\text{w}}_{\text{i}}}$ and $\bar{\text{y}}=\frac{\sum {\text{w}}_{\text{i}}{\text{y}}_{\text{i}}}{\sum {\text{w}}_{\text{i}}}$ , ${\text{w}}_{\text{i}}$ is the reliability-based weighting of animal i and calculated as $\left({\text{Rel}}_{\text{xi}}\times {\text{Rel}}_{\text{yi}}\right)/\sqrt{{\text{Rel}}_{\text{xi}}\times {\text{Rel}}_{\text{yi}}}$ $\left(\text{Rel}={\text{Acc}}^{2}\right)\left(\text{Rel}={\text{Acc}}^{2}\right)$ , and ${\text{x}}_{\text{i}}$ and ${\text{y}}_{\text{i}}$ are the GEBV of trait x and y , respectively. The standard errors of the approximated genetic correlations were estimated as:

$$\text{SE}=\sqrt{\left(1-{\hat{\text{r}}}_{\text{g}\left(\text{xy}\right)}^{2}\right)/\left(\text{n}-2\right)},$$

where n is the number of animals with GEBV theoretical accuracy (for intercept and slope terms) greater than 0.30. The genetic correlations estimated between ENV and the approximated genetic correlation between traits were only calculated for the selected optimal ENV for each trait (chosen according to the three criteria previously mentioned). Genotype-by-environment interactions were considered large, moderate, or weak if the genetic correlations between environments were lower than 0.50, between 0.50 and 0.80, and higher than 0.80, respectively.

## Results

### Descriptive Statistics

The descriptive statistics after the QC are shown in Table 2. The number of observations ranged from 6,059 for WN to 172,984 for TNB. Systematic and random effects for each trait were defined and are described in Table 2. In summary, the effect of sex, birth parity, contemporary group (growth), and weaning age divided into different classes was used for MDP (four classes), BFT (five classes), WW (five classes), and OTW (four classes); farrow parity, contemporary group (reproductive), and farrowing age divided into 11 different classes were used for TNB, NBA, NBD, WN, and IWE; and seven classes were used for IBF. Among the random effects, animal additive genetic effects were considered for all traits, animal permanent environmental effects for TNB, NBA, NBD, and IWE, and common environment effects for MDP, BFT, WW, OTW, TNB, NBA, NBD, and IWE.

### Selection of Environmental Descriptor

#### Slope of Reaction Norm Model

Table 3 presents the descriptive statistics for the ENV selected for each trait and the variance component estimated for the additive genetic slope term (σ^2^_a1_) using RNM. For completeness, Supplementary Table S3 presents the variance components estimated for all “ENV × critical period × trait” studied. Among the selected ENV, RH was selected for five out of the 10 studied traits (MDP, BFT, NBD, WN, and WW). For the remaining traits (OTW, TNB, NBA, IBF, and IWE), MaxT was considered as the best ENV descriptor. The variance component estimates were partially consistent within trait, except for OTW considering a critical period average of 30 days (OTW_30), where the σ^2^_a1_ for RH and DewP were 94.28 and 89.05% lower than the higher σ^2^_a_ (THI), respectively. For OTW_30, MaxT and THI resulted in a similar σ^2^_a1_ (52.351 and 53.123 kg^2^). Meanwhile, when considering a critical period of, on average, 120 days for OTW (OTW_120) and despite the fact that MaxT also gave the highest σ^2^_a1_ (32.890 kg^2^), it was lower than in OTW_30, suggesting that a period of, on average, 30 days would be better to evaluate HTol for OTW. The same trend from OTW was observed for MDP and BFT, where the average of 30 days (MDP_30 and BFT_30, respectively) resulted in higher σ^2^_a1_ estimates. However, as opposed to OTW, higher σ^2^_a1_ was observed when considering RH as the climatic gradient for both MDP_30 and BFT_30 (1.230 and 1.025 mm^2^, respectively). A similar trend was observed between IBF and IWE when considering MaxT, resulting in the largest estimate of σ^2^_a1_ (4.505 and 0.9816 days^2^, respectively), and RH, resulting in the lowest σ^2^_a1_ (1.663 and 0.446 days^2^, respectively).

**TABLE 3:** Description of heritability estimates of each selected environmental variable for each trait.

Trait	Critical period	Environmental variable	Heritability				σ^b^_a_ slope (PSD)
			Min	Mean	Max	SD	
OTW	30 days prior to measurement date	MaxT	0.21	0.25	0.47	0.038	52.3510 (6.380)
MDP	30 days prior to measurement date	RH	0.27	0.29	0.31	0.011	1.2301 (0.576)
BFT	30 days prior to measurement date	RH	0.38	0.42	0.47	0.019	1.0250 (0.466)
TNB	20 days prior to breeding to 30 days into gestation	MaxT	0.09	0.11	0.12	0.008	0.2095 (0.053)
NBA	20 days prior to breeding to 30 days into gestation	MaxT	0.08	0.09	0.12	0.011	0.2727 (0.062)
NBD	20 days prior to breeding to 30 days into gestation	RH	0.04	0.06	0.09	0.009	0.0122 (0.004)
IBF	34 days prior to farrowing to weaning date	MaxT	0.03	0.04	0.10	0.017	4.5055 (1.170)
IWE	34 days prior to farrowing to weaning date	MaxT	0.04	0.05	0.08	0.011	0.9816 (0.216)
WN	34 days prior to farrowing to weaning date	RH	0.07	0.11	0.31	0.048	1.4734 (0.772)
WW	34 days prior to farrowing to weaning date	RH	0.05	0.08	0.26	0.036	0.7150 (0.367)

MDP, ultrasound muscle depth (mm); BFT, ultrasound backfat thickness (mm); WW, weaning weight (kg); OTW, off-test weight (kg); TNB, total number of piglets born; NBA, number of piglets born alive; NBD, number of piglets born dead; WN, number of piglets weaned; IWE, interval between wean to estrus (days); IBF, interval between farrows (days); MaxT, average of maximum daily temperature; RH, average of daily relative humidity; Min, minimum; Max, maximum; SD, standard deviation; PSD, posterior standard deviation.

Considering the traits TNB, NBA, NBD, WN, and WW, RH had the highest σ^2^_a1_ estimates for all three of them. For TNB, MaxT had the highest σ^2^_a1_ estimate (0.2095) and RH the lowest (0.1042). MaxT was the highest for NBA (0.2727) and DI the lowest (0.0364). The trait NBD had the lowest σ^2^_a1_ estimates compared to all the other studied traits, being RH the ENV with the highest σ^2^_a1_ (0.0122) and MaxT the lowest (0.0022), showing an opposite trend than TNB. Two critical time periods were evaluated for WN and WW considering 34 days prior to the farrow date (abbreviated as WN_fd and WW_fd, respectively) and 34 days prior to farrowing until weaning date (abbreviated as WN_wd and WW_wd, respectively), resulting in the largest estimates of σ^2^_a1_ considering the critical period until weaning date. For WN_fd and WN_wd, RH had the largest σ^2^_a1_ estimate (1.291 and 1.473, respectively), while the lowest was DI (0.719) for WN_fd and MaxT (0.4102) for WN_wd. The same pattern of WN was observed for WW, where for WW_fd and WW_wd the largest estimate of σ^2^_a1_ came from RH (0.373 and 0.715 kg^2^, respectively) and the lowest from DI (0.077 kg^2^) for WW_fd and MaxT (0.046 kg^2^) for WW_wd. In summary, RH (MDP, BFT, NBD, WN, and WW) and MaxT (OTW, TNB, NBA, IBF, and IWE) were the ENV yielding the highest estimates of the slope term. In addition, for OTW_30, both MaxT and THI could be recommended based on similar results.

#### Theoretical Accuracy of GEBV

Overall, the accuracy of GEBV was greater for the intercept than for the slope term. The largest accuracy for the intercept was observed for BFT_30 considering MaxT (0.7224) as the ENV, and the lowest was for WN_fd considering RH (0.3121) as the ENV. Considering the slope term, the highest accuracy was observed for MaxT (0.6375) in OTW_30 and the lowest was MinT (0.2547) in MDP_120. Within each trait, it was not possible to observe a clear pattern regarding the ranking of ENV, as it was variable depending on the trait analyzed. The accuracy estimates for each trait considering all ENV are shown in Supplementary Table S2, and the accuracy values of the recommended ENV for each trait are shown in Table 4.

**TABLE 4:** Accuracies (95% confidence interval) of genomic estimated breeding value for the reaction norm intercept and slope terms considering the recommended environmental variable.

Trait	Environmental variable	GEBV accuracies			
		Intercept		Slope	
		Average	95% CI	Average	95% CI
OTW_30	MaxT	0.6275	0.6270–0.6279	0.6347	0.6341–0.6351
MDP_30	RH	0.6557	0.6541–0.6574	0.3554	0.3539–0.3569
BFT_30	RH	0.7017	0.7000–0.7033	0.5772	0.5756–0.5787
WN_wd	RH	0.4398	0.4376–0.4420	0.3693	0.3671–0.3715
WW_wd	RH	0.4206	0.4194–0.4218	0.4726	0.4713–0.4739
TNB	MaxT	0.6562	0.6555–0.6568	0.4702	0.4696–0.4708
NBA	MaxT	0.6435	0.6429–0.6441	0.4694	0.4687–0.4700
NBD	RH	0.6144	0.6138–0.6150	0.4388	0.4381–0.4394
IBF	MaxT	0.5313	0.5306–0.5320	0.4587	0.4580–0.4594
IWE	MaxT	0.5669	0.5663–0.5676	0.4587	0.4581–0.4594

OTW_30, off-test weight (kg) considering an interval of 30 days; MDP_30, ultrasound muscle depth (mm) considering an interval of 30 days; BFT_30, ultrasound backfat thickness (mm) considering an interval of 30 days; TNB, total number of piglets born; NBA, number of piglets born alive; NBD, number of piglets born dead; IWE, interval between wean to estrus (days); IBF, interval between farrows (days); WN_wd, number of piglets weaned considering measurement until weaning date; WW_wd, weaning weight (kg) considering measurement until weaning date; MaxT, average of maximum daily temperature; RH, average of daily relative humidity; CI, confidence interval.

The critical period of 30 days, on average, presented higher accuracies than the critical period of 120 days, on average, for BFT, OTW, and MDP. For BFT_30, the estimated accuracy for the intercept term ranged from 0.6960 (THI) to 0.7161 (DewP), while for the slope it ranged from 0.4118 (DI) to 0.5772 (RH). A different pattern, with lower estimates was observed for BFT_120, ranging from 0.6855 (MaxT) to 0.7151 (MinT) for the intercept and from 0.4153 (MeanT) to 0.4677 (MinT) for the slope. For OTW_30, the accuracy ranged from 0.5985 (RH) to 0.7132 (MaxT) for the intercept and from 0.5562 (DewP) to 0.6375 (MaxT) for the slope term, while OTW_120 ranged from 0.6156 (DewP) to 0.7738 (MaxT) and from 0.4279 (MinT) to 0.0.4909 (THI) for the intercept and slope, respectively. For MDP_30, the accuracy estimates for the intercept were similar between ENV, with 0.6405 (THI) and 0.6629 (MaxT), for the lowest and the highest values, respectively. For the slope term of MDP_30, the accuracy of GEBV ranged from 0.3103 (DI) to 0.3554 (RH). Considering MDP_120, the estimates ranged from 0.6330 (THI) to 0.0.6759 (MaxT) for the intercept and from 0.2547 (MinT) to 0.3209 (RH).

For TNB, NBA, and NBD, the accuracy estimates of GEBV for the intercept and slope terms had similar values between traits and ENV. The accuracy of GEBV for the intercept term ranged from 0.6473 (THI) to 0.6562 (MaxT), from 0.6287 (MeanT) to 0.6463 (MinT), and from 0.6128 (THI) to 0.6239 (MeanT) for TNB, NBA, and NBD, respectively. For the slope, the GEBV accuracies ranged from 0.4201 (MeanT) to 0.4702 (MaxT), from 0.3809 (RH) to 0.4694 (MaxT), and from 0.4090 (MaxT) to 0.4388 (RH) for TNB, NBA, and NBD, respectively. Regarding IBF, GEBV accuracy ranged from 0.4776 (DewP) to 0.5776 (THI) and from 0.3208 (MeanT) to 0.4587 (MaxT) for the intercept and slope terms, respectively. For IWE, the GEBV accuracies ranged from 0.5084 (DI) to 0.4049 (MaxT) and from 0.3372 (MinT) to 0.4587 (MaxT), respectively, for the intercept and slope terms.

Finally, considering the critical period of 34 days prior to farrowing date and 34 days prior to farrowing date until weaning date for WN and WW, respectively, more accurate GEBVs were observed for the intercept term as for the other discussed traits. For WN_fd, GEBV accuracies ranged from 0.3121 (RH) to 0.4710 (DI) and from 0.2735 (MinT) to 0.3475 (RH) for the intercept and slope terms, respectively. For WN_wd, the estimates ranged from 0.3253 (MaxT) to 0.4680 (RH) and from 0.2792 (MinT) to 0.3693 (RH) for the intercept and slope terms, respectively. The only trait with higher GEBV accuracy for the slope than intercept was for WN_fd, where an accuracy of 0.3121 for the intercept and 0.3475 for the slope was observed for RH. Considering WW_fd, the estimated accuracy of GEBV ranged from 0.4144 (THI) to 0.5172 (RH) and from 0.3373 (MaxT) to 0.4126 (RH) for the intercept and slope terms, respectively. For WW_wd, the estimates for the intercept and slope ranged from 0.4215 (MinT) to 0.5895 (THI) and from 0.3703 (MinT) to 0.4726 (RH), respectively. As observed for the first criterion, RH (BFT, MDP, NBD, WN, and WW) and MaxT (IBF and IWE) were the ENV yielding the highest theoretical accuracy of GEBV. For the other traits not previously mentioned, more than one climatic variable could be recommended (RH, THI, and MaxT for OTW, MaxT and RH for TNB, and MaxT and MeanT for NBA).

#### Deviation of GEBV per Environmental Variable

High GEBV standard deviations in a certain environmental value means that the identification of animals with the highest and lowest genetic values will be more evident. In this regard, the deviation estimates were similar within each value of ENV within trait. The deviation estimate followed a pattern similar to that found for the RN slope and accuracy of GEBV estimates, being the ENV with the largest deviations the same, in most cases, as those that yielded higher σ^2^_a_ and accuracy results. For OTW_30, OTW_120, TNB, NBA, IBF, and IWE, MaxT yielded the greatest deviation in most of the five categories (each category represents the value allocated in the 0th, 25th, 50th, 75th, and 100th position in each ENV). For the MDP_30, MDP_120, BFT_30, NBD, WN_wd, WN_fd, and WW_wd, the ENV based on RH yielded the higher deviation in most of the ENV values. Lastly, for the remaining traits, there was no absolute ENV that yielded higher deviations. In the case of BFT_120, MaxT, MinT, and DI yielded the highest values, and for WW_fd, the highest values were for RH, MeanT, and MinT. A complete estimation of the deviation in each “ENV × critical period × trait” combination is shown in Supplementary Table S4.

ptA summary of the results of the three criteria presented above is shown in Table 4 as well as the recommended ENV and critical period for each trait. In brief, the critical period of an average of 30 days yielded higher estimates of σ^2^_a_ and accuracy and deviation of GEBV per ENV. Additionally, the critical period ranging from 34 days prior to farrowing date until weaning date yielded the highest estimation of the three criteria. Moreover, the recommended ENVs for OTW_30 were THI and MaxT, for BFT_30, MDP_30, NBD, WN_wd, and WW_wd was RH, and for TNB, NBA, IBF, and IWE was MaxT. Subsequent results will be presented only for the recommended ENV and critical periods.

### Heritability Estimates

**FIGURE 1:**
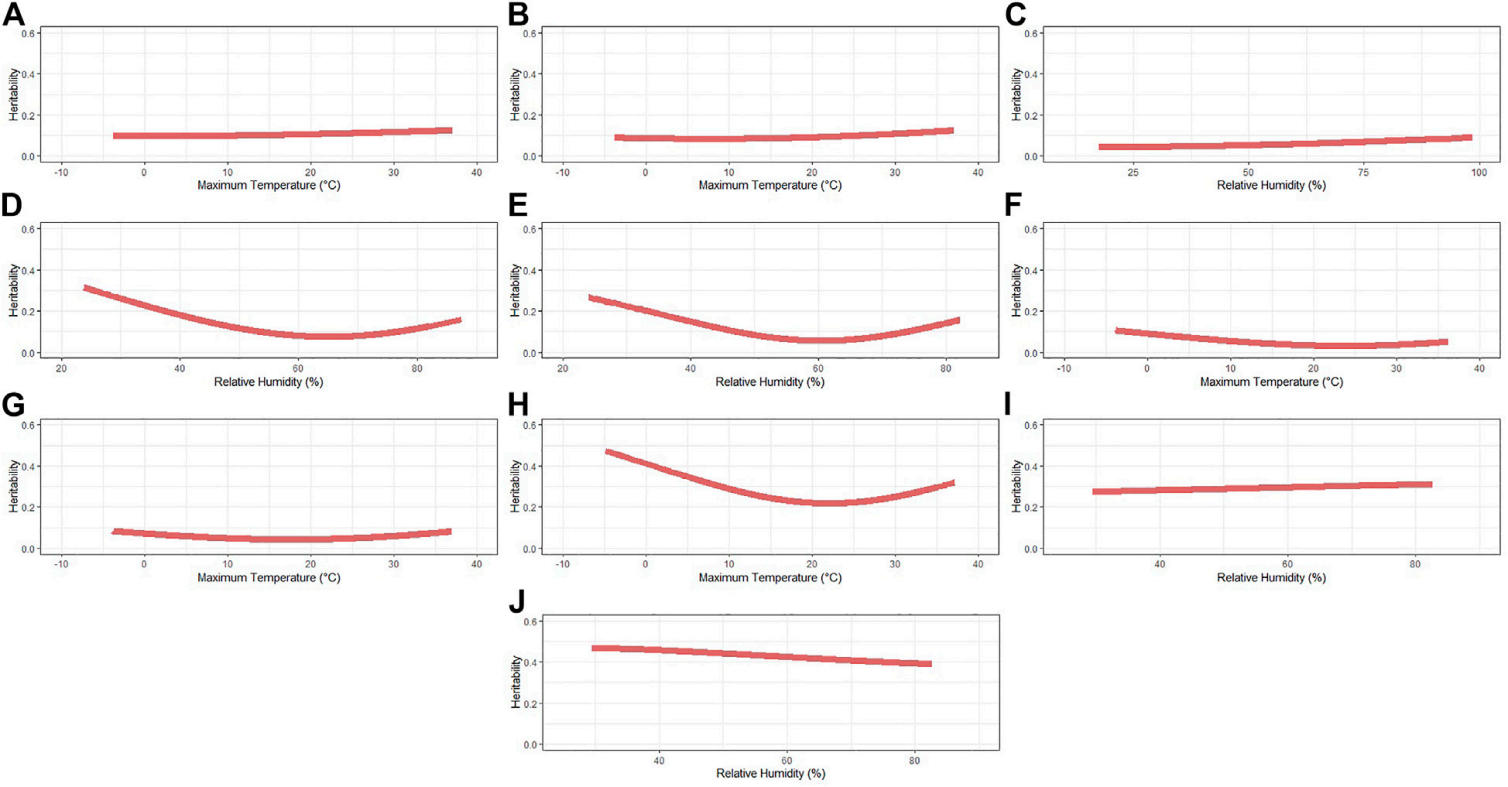
Heritability estimates for each recommended environmental variable and critical period for each studied trait. **(A)** Total number of piglets born. **(B)** Number of piglets born alive. **(C)** Number of piglets born dead. **(D)** Number of piglets weaned considering measurement until weaning date. **(E)** Weaning weight (kg) considering the measurement of records until weaning date. **(F)** Interval between wean to estrus (days). **(G)** Interval between farrows (days). **(H)** Off-test weight (kg) considering an interval of 30 days. **(I)** Ultrasound muscle depth (mm) considering an interval of 30 days. **(J)** Ultrasound backfat thickness (mm) considering an interval of 30 days.

The heritability (*h* ^2^) estimates across the range of the selected ENV for each trait are shown in Table 3 and the complete pattern in Figure 1. The average *h* ^2^ ranged from 0.04 (IBF) to 0.42 (BFT_30). A similar pattern was observed for TNB (from 0.09 to 0.12), NBA (from 0.08 to 0.12), and NBD (from 0.04 to 0.09), where a slight increase in *h* ^2^ is observed as the ENV values increase. The traits OTW_30 (from 0.21 to 0.47), WN_wd (from 0.07 to 0.31), and WW_wd (from 0.05 to 0.26) also had a similar pattern, with higher *h* ^2^ in extreme ENV values and lower estimates closer to the mean. An opposite pattern of average *h* ^2^ was observed for MDP_30 (from 0.27 to 0.31) and BFT_30 (from 0.38 to 0.47). As the values of ENV increased, the *h* ^2^ for MDP_30 also increases, and for BFT_30 it decreases. The heritability estimates within each trait (*i*.*e*., considering all the ENV and critical periods evaluated) had similar patterns and are shown in Supplementary Table S5.

### Reaction Norms and Re-ranking Across Environments

**FIGURE 2:**
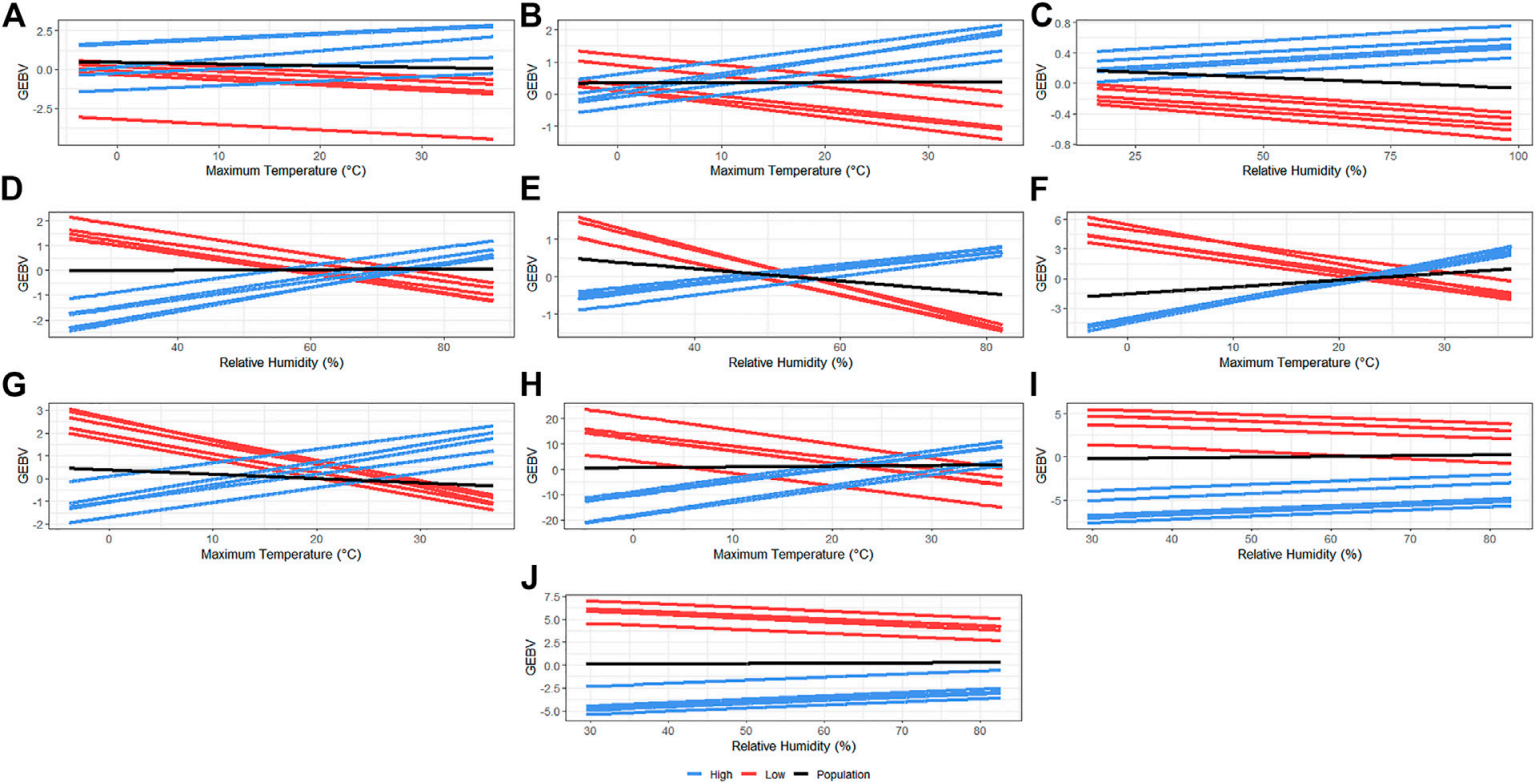
Genomic estimated breeding values for each recommended environmental variable and critical period for each studied trait considering five most heat-tolerant and five most heat-susceptible sires. The black line indicates the population trend, the blue lines indicate the most tolerant sires, and the red line indicates the most susceptible sires. **(A)** Total number of piglets born. **(B)** Number of piglets born alive. **(C)** Number of piglets born dead. **(D)** Number of piglets weaned considering measurement until weaning date. **(E)** Weaning weight (kg) considering measurement until weaning date. **(F)** Interval between wean to estrus (days). **(G)** Interval between farrows (days). **(H)** Off-test weight (kg) considering an interval of 30 days. **(I)** Ultrasound muscle depth (mm) considering an interval of 30 days. **(J)** Ultrasound backfat thickness (mm) considering an interval of 30 days.

Sires were selected to have at least 30 offspring so that the phenotypes were distributed under a large range in ENV (*i*.*e*., records of daughters could not be concentrated under the comfortable environmental gradients). The reaction norms for the slope term of the additive genetic effect of the five most HT and the five most heat-susceptible (HSusc) sires are reported in Figure 2. Re-rankings of animals were clearly observed for NBA, WN_wd, WW_wd, IWE, IBF, and OTW_30, while no clear re-ranking was observed for NBD, MDP_30, and BFT_30. Meanwhile, there was a trend of re-ranking observed for TNB at the best environmental conditions. For TNB (Figure 2A), HT sires appear to have higher performance than the HSusc sires even when the temperature increases. For NBA (Figure 2B), IWE (Figure 2F), IBF (Figure 2G), and OTW_30 (Figure 2H), a clear distinction between groups was observed, with HT sires performing better in the highest temperatures, while the HS performed better in the lower temperatures. For WN_wd (Figure 2D) and WW_wd (Figure 2E), the HT sires were observed to have better performance under environments with higher humidity, while the HSusc sires had a better performance under drier environments. For MDP_30 (Figure 2I) and BFT_30 (Figure 2J), no re-ranking was observed. For all traits, even though some of them do not have a clear re-ranking, it is possible to observe that there are groups of animals performing better than the average population performance (Figure 2, black lines) in challenging environments.

In addition, the Spearman (rank) correlations are shown in Supplementary Figure S2 for combinations of intercept and slope terms between each pair of traits. The greatest rank correlations were between TNB and NBA, where between intercepts was equal to 0.917 and between slopes was equal to 0.838, indicating that the GEBV of TNB tends to increase when the GEBV of NBA increases. All other trait combinations had moderate to low rank correlations. Between MDP_30 and OTW_30, IBF and TNB, IBF and NBA, and between MDP_30 and TNB, the slope terms were equal to 0.216, 0.155, 0.188, and 0.113. Negative rank correlations between the slope terms were observed for WN-wd and TNB (−0.196), WN_wd and NBA (−0.185), BFT_30 and WW_wd (−0.149), and IWE and WW_wd (−0.141). The remaining rank correlations between trait combinations for the intercept and slope terms had low rank correlations and are presented in Supplementary Figure S2.

### Genetic Correlation Between Environments

**FIGURE 3:**
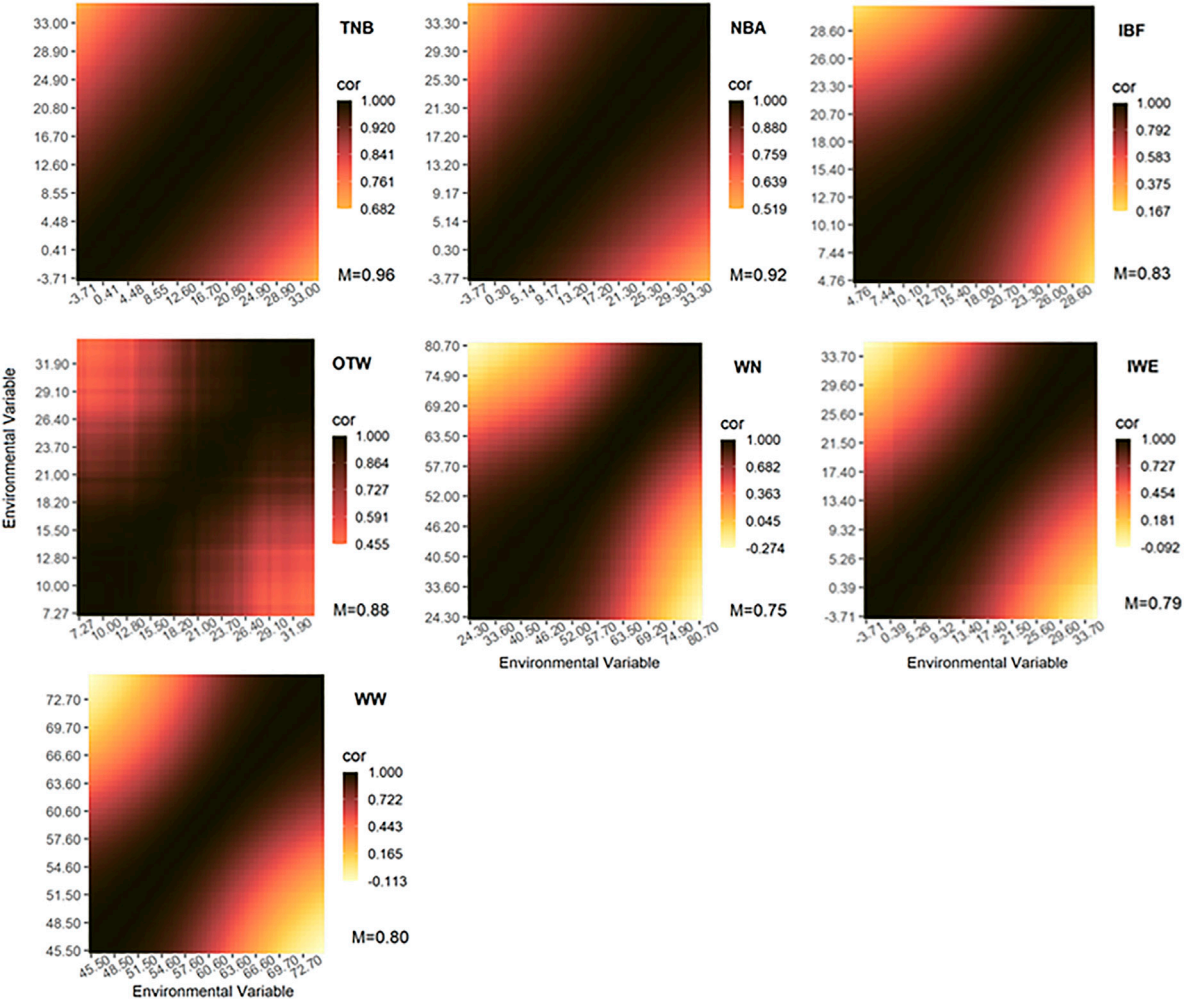
Genetic correlations across environmental gradients using the recommended environmental variable and critical period for each studied trait. The Pearson correlation coefficient (cor) are represented by colors with the mean values shown below. TNB, total number of piglets born; NBA, number of piglets born alive; WN, number of piglets weaned considering the measurement of records until weaning date; WW, weaning weight (kg) considering measurement until weaning date; IWE, interval between wean to estrus (days); IBF, interval between farrows (days); OTW, off-test weight (kg) considering an interval of 30 days.

The genetic correlations of additive genetic effects across environmental gradients are shown in Figure 3 (for weak and moderate correlations) and Supplementary Figure S1 (for strong correlations). All estimated genetic correlations between environments considered just ENV values ranging from the 10th to the 90th percentiles, therefore excluding extreme ENV values. The exclusion of the extreme values was done to remove ENV with a low number of records, which might affect the estimation of the parameters at the extremes. As expected, the genetic correlation decreased gradually for greater differences among the environmental gradients. The lowest genetic correlation was observed for WN_wd (Figure 3; average = 0.75), even reaching negative values (-0.274). In addition to WN_wd, IBF (Figure 3; average = 0.79), WW_wd (Figure 3; average = 0.80), and IWE (Figure 3; average = 0.83) seem to be largely affected by G×E interaction. Moderate interactions were observed for OTW_30 (Figure 3; average = 0.88), NBA (Figure 3; average = 0.92), and TNB (Figure 3; average = 0.96). The highest genetic correlations were observed for NBD (Supplementary Figure S1; average = 0.98), MDP_30 (Supplementary Figure S1), and BFT_30 (Supplementary Figure S1), with both of the latter having an average of 0.99. Therefore, we concluded that WN_wd, IBF, WW_wd, and IWE are largely and OTW_30, NBA, and TNB are moderately affected by the G×E interaction. However, NBD, MDP_30, and BFT_30 were only mildly affected by G×E interaction.

### Approximated Weighted Genetic Correlations Between Traits

**FIGURE 4:**
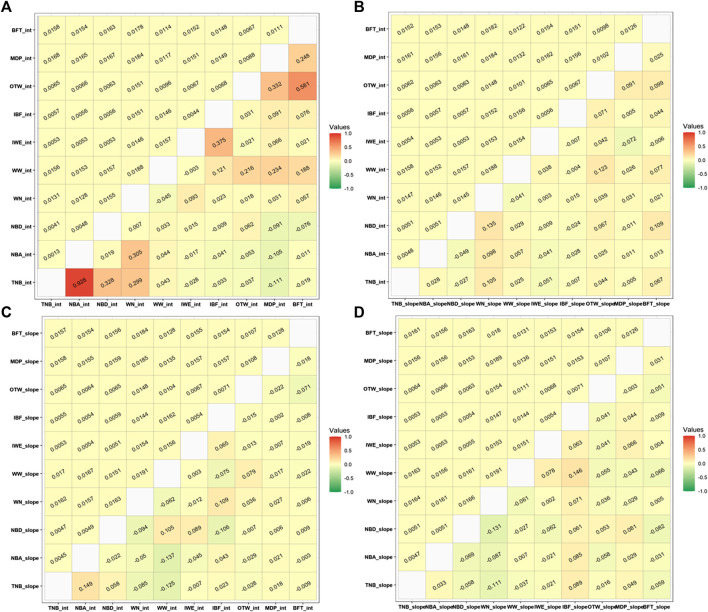
Approximated Pearson genetic correlations (lower) and standard errors (upper) among **(A)** intercept terms, **(B)** intercept and slope, **(C)** slope and intercept, and **(D)** slope terms of the recommended environmental variable and critical period for each studied trait. TNB, total number of piglets born; NBA, number of piglets born alive; NBD, number of piglets born dead; WN, number of piglets weaned; WW, weaning weight (kg); IWE, interval between wean to estrus (days); IBF, interval between farrows (days); OTW, off-test weight (kg); MDP, ultrasound muscle depth (mm); BFT, ultrasound backfat thickness (mm). For OTW, MDP, and BFT, we considered a critical period of an average of 30 days before measurement date. For WN and WW, we considered a critical period from 34 days prior to farrowing up to weaning date. For TNB, NBA, and NBD, we considered a critical period of 20 days prior to breeding to 30 days into gestation. For IBF and IWE, we considered a critical period of 34 days prior to farrowing to weaning date.

Individuals with GEBV theoretical accuracies higher than 0.30 were used to calculate the weighted Pearson correlation among the RN intercepts and slopes between all studied traits considering the recommended ENV for each trait. The minimum number of selected animals and the lowest average accuracies were 5,718 and 0.4224 (95% CI of 0.4210–0.4236), respectively (Supplementary Table S6). The approximate genetic correlations between intercepts, intercept and slope, slope and intercept, and slope and slope for each pair of traits (considering the recommended ENV for each trait) are shown in Figure 4.

The greatest genetic correlations were observed between the intercept terms. The greatest positive correlations were observed between TNB and NBA, with 0.928, and between BFT_30 and OTW_30 (0.581) for the RN intercept. A moderate genetic correlation was also observed between TNB and NBD (0.328), TNB and WN_wd (0.305), and NBA and WN_wd (0.305). Moderate genetic correlations between intercepts were also observed between IBF and IWE (0.375), MDP_30 and OTW_30 (0.332), and BFT_30 and MDP_30 (0.248). For WW_wd, moderate to low genetic correlations were observed with IBF (0.121), OTW_30 (0.216), MDP_30 (0.234), and BFT_30 (0.188). The combinations for the remaining traits for the intercept term had low genetic correlations and are shown in Figure 4A.

Approximate genetic correlations between the intercept and slope terms indicate what happens to the performance of an animal for a specific trait when another trait is expressed under challenging heat conditions. In this matter, the greatest positive correlation was between TNB slope and NBA intercept (0.149) and between NBD intercept and WN slope (0.135). A similar positive correlation was also observed between WW intercept and OTW slope (0.123) and between the pairs of BFT slope with NBD intercept and IBF intercept with WN slope (both equal to 0.109). The WN slope and TNB intercept and the NBD slope and WW intercept had the same correlation value of 0.105. The greatest negative correlation was observed between the NBA slope and the TNB slope with WW intercept (−0.137 and −0.125, respectively). All other combinations between intercept and slope had values lower than 0.100 (Figure 4B,C).

The smallest genetic correlations were observed between the slope terms. This correlation indicates the relationship between the traits under a stressful environmental condition. The greatest positive slope × slope genetic correlation was observed for WW_wd and IBF (0.146). A negative correlation of -0.131 was observed for NBD and WN_wd and of -0.111 between WN_wd and TNB. All the other trait combinations had correlations under the absolute value of 0.100 and are shown in Figure 4D.

## Discussion

A set of comprehensive analyses to reveal the genetic background of HTol based on routinely measured phenotypic records and publicly recorded weather variables was performed in this study.

### Environmental Variable Selection

As indicated in the “Materials and Methods” section, three different metrics were used as criteria for the selection and recommendation of ENV and critical period to evaluate HTol in pigs from the Large White breed (one of the main maternal-line breeds). The first criterion, based on the ENV yielding the higher G×E interaction measured by the σ^2^_a1_ term, was also used by other studies evaluating HS effects in reproductive and carcass traits in pure breeds and crossbred animals (Tiezzi et al., 2020; Usala et al., 2021). The second criterion was based on the theoretical accuracy of GEBV. Therefore, the climatic metric recommended had to not only have the strongest G×E but also provide more reliable GEBV estimates. Furthermore, the third criterion was based on the ENV yielding the greatest deviation of GEBV within the ENV value. The latest mentioned criterion was considered because, in this study, the goal was to use an ENV that allows a clear distinction (*i*.*e*., higher dispersion of breeding values) between HT and HSusc animals. A large variability in GEBV (*i*.*e*., higher standard deviation of GEBV within each ENV value) allows better discrimination of the animals, which facilitates the selection of the desired animals. Another metric that could also be evaluated was the deviance information criterion (DIC). However, in this study, the DIC was not included as another criteria for ENV selection due to criticisms found in the literature, such as the lack of consistency, weak theoretical justification, and overfitting (Spiegelhalter et al., 2014; Clarke and Clarke, 2018; Salles et al., 2019).

Climate records from the National Climatic Data Center weather stations were used to describe the environmental conditions experienced by the sows at different stages of ovulation, pregnancy, and post-pregnancy. Weather stations used in the current study were on average within 30 km from the farm. Freitas *et al*. (2006) estimated a correlation of 0.9 between on-farm weather data and weather station data even for weather stations more than 300 km away from the farm. Although these climate records are usually publicly available and have been successfully used in other worldwide studies focusing on sow HTol for different reproduction, growth, and carcass traits (Tummaruk et al., 2010; Wegner et al., 2014; Tiezzi et al., 2020; Usala et al., 2021), we acknowledge that the collection of within-barn environmental measurements is important and should be done when possible. However, the variables needed for this study were not available for this current study. It is also worth noting that, although there was a large variability in the climatic variables used in this study, they might not be representative of all the possible range of climatic conditions. Therefore, additional studies should be performed in other populations (*e*.*g*., breeds) and geographical regions to confirm our results.

In the current study, acute HS had greater impact on OTW, BFT, and MDP than chronic HS, which suggests that, during the period of chronic HS, the animal might be able to recover from the thermal stress suffered. For these traits (OTW, BFT, and MDP), the variance components for the slope term were higher considering a period of 30 days before measurement than 120 days. The GEBV accuracy estimates were also greater for the acute HS. For WW and WN, the period ranging from the last stage of gestation (around day 80) until the weaning date resulted in higher G×E interaction and GEBV accuracies. Therefore, the time period ranging from the last stage of gestation until weaning date is the recommended critical period to evaluate HT for WW and WN (referred here as WW_wd and WN_wd, respectively).

Several studies implemented different climatic variables as the environmental gradient covariate—for instance, some studies used heat load calculated as THI greater than 70, temperature, humidity, or THI (Fragomeni et al., 2016; Tiezzi et al., 2020; Usala et al., 2021). However, reports of the best environmental metric to be used when evaluating HTol in pigs from the maternal line are scarce. Therefore, we evaluated several ENV based on temperature and humidity to achieve a recommendation for each of the evaluated traits. The use of an index-based environmental variable is another way of carrying out the analyses, and it has been done in plants (*e*.*g*., Resende et al., 2020). However, in the current study, single ENV was used as environmental gradient. The use of RH as the climatic variable is the recommendation for NBD, WW_wd, WN_wd, MDP_30, and BFT_30. Maximum temperature is the recommended ENV for TNB, NBA, IBF, IWE, and OTW_30. Even though for OTW_30 the use of MaxT was recommended based on our findings, THI also resulted in a very similar G×E interaction. This choice was based on THI development as most THI are originally related to the body temperatures of cattle exposed to HS and have been adapted to other species or populations (Ingraham et al., 1979; Buffington et al., 1981; Bohmanova et al., 2007). Therefore, the use of THI in pigs is viewed with caution since different physiological and anatomical characteristics (*i*.*e*., rumen, ability to sweat, body mass, *etc*.) are found between cattle and swine that directly influence thermotolerance. For OTW, a high (0.79) rank correlation between GEBV estimates using THI and MaxT was observed. Therefore, due to the caution in interpreting the THI values for swine and the high rank correlation between THI with MaxT, MaxT was recommended as the ENV to evaluate HTol in maternal-line pigs for OTW. For five out of the 10 traits evaluated, the ENV selected (Table 5) were built upon RH as climate variable and the other five used MaxT. The preponderance of RH, rather than other climatic variables, in challenged sows was reported by Tummaruk *et al*. (2010) and Tiezzi *et al*. (2020) who studied reproductive performance under HS conditions. This finding might indicate that the barn cooling systems can better mitigate the impact of temperature than RH.

**TABLE 5:** Summary and recommendation of the three criteria evaluated of each environmental variable for each trait.

Traits	σ^2^_a_ slope	GEBV accuracies	Deviation of GEBV per ENV	Measurement interval	Recommended ENV
OTW_30	THI and MaxT	MaxT	MaxT	30-day interval	MaxT
OTW_120	MaxT	RH and THI	MaxT		
BFT_30	RH	RH	RH	30-day interval	RH
BFT_120	RH	RH	MaxT		
MDP_30	RH	RH	MaxT and RH	30-day interval	RH
MDP_120	RH	RH	MinT and RH		
TNB	MaxT	MaxT and RH	MaxT and RH	No critical period evaluated	MaxT
NBA	MaxT	MaxT and MeanT	MaxT and RH	No critical period evaluated	MaxT
NBD	RH	RH	RH	No critical period evaluated	RH
IBF	MaxT	MaxT	MeanT and MaxT	No critical period evaluated	MaxT
IWE	MaxT	MaxT	MaxT	No critical period evaluated	MaxT
WN_wd	RH	RH	RH	Until weaning date	RH
WN_fd	RH	RH	RH		
WW_wd	RH	RH	RH	Until weaning date	RH
WW_fd	RH	RH	RH		

OTW_30, off-test weight (kg) considering an interval of 30 days; OTW_120, off-test weight (kg) considering an interval of 120 days; MDP_30, ultrasound muscle depth (mm) considering an interval of 30 days; MDP_120, ultrasound muscle depth (mm) considering an interval of 120 days; BFT_30, ultrasound backfat thickness (mm) considering an interval of 30 days; BFT_120, ultrasound backfat thickness (mm) considering an interval of 120 days; TNB, total number of piglets born; NBA, number of piglets born alive; NBD, number of piglets born dead; IWE, interval between wean to estrus (days); IBF, interval between farrows (days); WN_wd, number of piglets weaned considering measurement until weaning date; WN_fd, number of piglets weaned considering measurement until farrow date; WW_wd, weaning weight (kg) considering measurement until weaning date; WW_fd, weaning weight (kg) considering measurement until farrow date; MaxT, average of maximum daily temperature; MinT, average of minimum daily temperature; MeanT, average of mean daily temperature; DewP, average of daily dew point; RH, average of daily relative humidity; DI, average discomfort index; THI, average temperature–humidity index; ENV, environmental variable.

### G×E and Genetic Parameters

#### Heritability Estimates

Genomic RNM were used in this study for the estimation of genetic parameters, including additive genetic variance for the trait performance in a standard environment (σ^2^_a0_, intercept term) as well as the additive genetic variance for the tolerance to a given environmental stressor (σ^2^_a1_, slope term). The additive genetic variance estimates obtained agree with the estimates by Chen *et al*. (2021) when evaluating HT based on CGe in the same population. The heritability estimates are in the same range with the ones reported by Tiezzi *et al*. (2020), using the average RH before conception (for TNB) or the average THI index during the pregnancy of sows (for NBA). Chen *et al*. (2021), using CGe, also found a similar *h* ^2^ for TNB, NBA, WW, and MDP. A higher average *h* ^2^ for NW was estimated using RH as the environmental gradient when compared to CGe (Chen et al., 2021).

As described by Usala *et al*. (2021), the use of RNM to assess the environmental effect on animal fertility, conformation, or carcass quality traits is less common in pigs when compared to dairy and beef cattle (Meyer, 2000; Coffey et al., 2002; Bradford et al., 2016; Ansari-Mahyari et al., 2019). In pigs, the genetic component for HTol is usually calculated as a function of heat load on growth traits or carcass weight (Zumbach et al., 2008; Fragomeni et al., 2016). Estimating genetic parameters for HTol in crossbred swine carcass traits, Usala *et al*. (2021) also observed a high average *h* ^2^ for BFT and MDP. The heritability estimated for the beginning of the curve (assumed as a comfortable condition compared to the right extreme values) was also a trend observed by Tiezzi *et al*. (2020) for TNB and NBA. Silva et al. (2014) also reported a higher *h* ^2^ for TNB in contemporary groups with better performance, and a similar trend was observed by Fragomeni *et al*. (2016) when assessing HTol based on the body weight of purebred Duroc individuals. The use of quadratic functions is another alternative to analyze the data. However, considering the biological aspect of heat stress, the use of quadratic or higher-order functions can compromise the interpretation of results (*e*.*g*., using a quadratic function could mean that, when temperature increases, the impact of heat stress would be higher, but after a certain point, this impact would be favorable even though the temperature continues to increase).

#### Genetic Correlations Across Environments

The most common thresholds recommended for considering the presence of G×E interaction are based on genetic correlations across the range of ENV lower than 0.70 (Mulder and Bijma, 2007) or 0.80 (Hayes et al., 2016). In this study, only NW, WW, and IBF presented a G×E interaction considering an average threshold of 0.80. However, despite the fact that some traits had an average correlation value higher than the threshold correlation of 0.80, the minimum values found for TNB, NBA, IWE, and OTW were lower than this threshold. Therefore, we suggest that moderate G×E interactions were observed in such traits. For NBD, MDP, and BFT, both average and minimum values are above the 0.80 threshold, indicating a low G×E interaction across ENV values, which is also supported by the RN of GEBV for the mentioned traits (Figures 2C,I,J). Similar values of genetic correlations across ENV were found by Tiezzi *et al*. (2020) for TNB and NBA, in which the correlation between RH values reached 0.78 and that between THI reaching 0.83, respectively. Additionally, Chen *et al*. (2021) observed similar average values when considering CGe as the environmental covariate for BFT. However, differently from Chen *et al*. (2021), in the current study, stronger G×E interactions were observed for WN and moderate for OTW. Usala *et al*. (2021) observed lower values for the genetic correlations in crossbred animals (0.89 and 0.50 for BFT and loin depth, respectively, using RH). These results suggest that the G×E interaction is not only population dependent but also dependent on the environmental covariate used. It is important to note that the estimates of G×E might not reflect the exact environmental conditions experienced by the animals because public weather station data may not precisely represent within-barn environmental conditions. Therefore, further studies should consider in-barn recorded environmental data to validate the results.

Visualizing more HT animals can be facilitated using RN plots (Figure 2). Individuals with better performance under HS conditions can be selected and used within a breeding scheme, provided that they are not under-performing under thermoneutral conditions (*i*.*e*., comfortable and controlled climatic conditions). Except for TNB, NBD, MDP, and BFT, the best-performing animals under challenging environmental conditions (*i*.*e*., high humidity or temperature) are under-performing in comfortable conditions. The identification of these animals (or their descendants) means that the use of their genetic resources will be better applied on farms experiencing high RH or temperature values. For TNB and NBD, the most HT animals performed better under both comfortable and challenging conditions, and the opposite trend occurred with MDP and BFT, where the more HSusc animals outperformed the most HT across the entire ENV range. These results agree with the G×E interaction (Figures 3C,I,J), where high correlations were found for NBD, MDP, and BFT.

#### Approximated Genetic Correlations Between Traits

The highest positive genetic correlations observed between TNB and NBA intercept terms were also observed by Serenius *et al*. (2004) in Finnish Large White pigs. Serenius *et al*. (2004) estimated the genetic correlation for first farrowing interval with TNB, NBA, and WN to be higher (0.130, 0.160, and 0.140, respectively) than the values found in this study, where the correlation between IBF intercept and TNB, NBA, and WN intercept had negative values (−0.033, −0.041, and −0.009). Moderate correlations between OTW, MDP, and BFT were observed in this study and were also reported by Chen *et al*. (2021) and Jiao *et al*. (2014). The genetic correlations among slopes for different traits were low to moderate, which indicate that HTol based on different traits could result in the selection of different animals. These results are important to the industry as they indicate that pigs should be selected for HTol defined based on multiple traits and potentially combined in a selection sub-index. However, as there are no gold-standard values (*e*.*g*., true breeding values), the fact that the breeding values for HTol based on alternative traits are different or that re-ranking occurred cannot be used for choosing the best environmental variable for each trait. In addition, the fact that these correlations between slopes are low to moderate indicates the need to evaluate traits in different environments. These findings contribute to improving selection strategies under HT and suggest that different animals would be selected depending on the trait evaluated. Selection can be achieved by selecting for all traits, especially when including the traits in a selection sub-index, according to the desired breeding goal.

### Reaction Norm Gradient: Comparison Between Contemporary Group Effect and Environmental Variable

Different environmental gradients for the RN have been used over time (CGe: Li and Hermesch, 2016; Song et al., 2020; Chen et al., 2021 and climatic variables: Fragomeni et al., 2016; Tiezzi et al., 2020; Usala et al., 2021). However, the feasibility of using the average CGe or climatic variables as the environmental gradient for assessing G×E interactions is unknown. In this regard, Chen *et al*. (2021), using the same population and seven traits also used in the current study (OTW, MDP, BFT, TNB, NBA, WN, and WW), calculated the GEBV of animals using CGe as the environmental gradient. In general, the estimates based on the recommended ENV of the current study resulted in higher theoretical accuracies of GEBV, except for MDP, where the accuracy based on CGe was 0.580 (*i*.*e*., indicator of G×E interaction), while the accuracy based on the recommended ENV was 0.350 for the slope term. A Spearman (rank) correlation between the GEBV of individuals regressed on the recommended ENV and CGe shows a negative moderate rank correlation for BFT (−0.57), WW (−0.47), and MDP (−0.31) and a low positive rank correlation for the other traits (Supplementary Table S7). These results suggest that, despite being used and historically providing reliable breeding value estimates (Song et al., 2020; Chen et al., 2021), the use of CGe accounts for additional variability, including nutrition practices and management. This might not be the interest when evaluating HTol. Therefore, as suggested, the use of ENV might be more useful when the objective is to evaluate the breeding values of animals under challenging climatic stress.

## Conclusion

The genetic and genomic background of HTol was comprehensively explored in the present study for various reproduction, growth, and body composition traits measured in pigs from the Large White breed (maternal line). Moreover, the use of different critical periods and climatic variables defined based on public weather station records was evaluated. Our results indicate that evaluating HS in the period of 30 days before the measurement date provided the best estimates of G×E for OTW, MDP, and BFT. Additionally, the critical period ranging from the last trimester of gestation until weaning provided better estimates for WN and WW. From the climatic variables evaluated, the average RH (for NBD, WN, WW, MDP, and BFT) and MaxT (for TNB, NBA, IWE, IBF, and OTW) are the recommended variables to evaluate HS based on the mentioned traits. Large (WN, WW, IBF, and IWE) and moderate (OTW, TNB, and NBA) G×E interactions were observed for most of the studied traits, while other traits (MDP, BFT, and NBD) are less impacted by heat stress, showing a weaker evidence of G×E. The low to moderate genetic correlations between HTol breeding values based on different traits indicate that a selection sub-index combining the breeding values for the slopes of multiple traits might be needed for identifying the most HT animals throughout their entire productive life. The current study also demonstrates that HTol is heritable, and genetic progress can be achieved through direct genetic and genomic selection. Therefore, selecting for improved HTol based on reproductive, growth, and body composition performance and public weather station data is feasible in swine. Future research using within-barn environmental records will be performed next.

## Data Availability

The data analyzed in this study is subject to the following licenses/restrictions: The phenotypic and genomic data used in this study are a property of the industry partner that contributed to the study and therefore are not readily available due to its commercially sensitivity. Requests to access these datasets should be directed to Yijian Huang, yhuang@smithfield.com.
