# Supplementary figures and images for: Definition of Environmental Variables and Critical Periods to Evaluate Heat Tolerance in Large White Pigs Based on Single-Step Genomic Reaction Norms
Source: Front Genet. 2021 Nov 23;12:717409. doi: 10.3389/fgene.2021.717409 (PMC8650309; doi:10.3389/fgene.2021.717409)

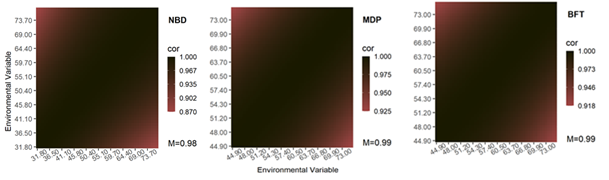

Supplement: Supplementary file 1 [file Image1.JPEG]

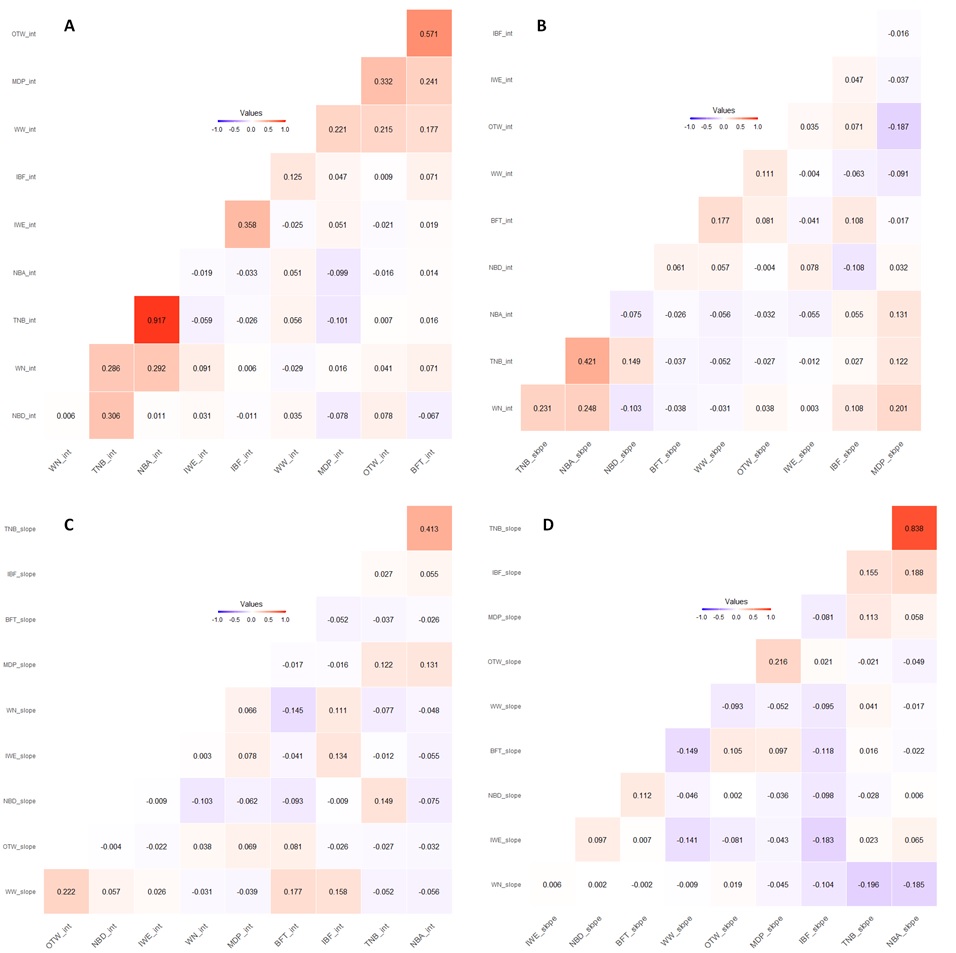

Supplement: Supplementary file 3 [file Image2.JPEG]
