# Supplementary material for: Definition of Environmental Variables and Critical Periods to Evaluate Heat Tolerance in Large White Pigs Based on Single-Step Genomic Reaction Norms
Source: Front Genet. 2021 Nov 23;12:717409. doi: 10.3389/fgene.2021.717409 (PMC8650309; doi:10.3389/fgene.2021.717409)
Supplement: Supplementary file 2 [file DataSheet1.zip › Tables 1 - 2.DOCX]

**Definition of environmental variables and critical periods to evaluate heat tolerance in Large White pigs based on single-step genomic reaction norms**

***Freitas et al*.**

**Contents:**

**Table S1.** Description of the environmental variables (ENV) used for number of piglets born total.

**Table S2.** Accuracies (95% confidence interval) of genomic estimated breeding value for the reaction norm intercept and slope terms considering all environmental variable.

**Table S3.** Estimation of variance components for the intercept and slope for all environmental variables and critical periods for each studied trait.

**Table S4.** Deviation of genomic estimated breeding value (GEBV) in five different environmental values.

**Table S5.** Description of heritability estimates for all analyzed environmental variable for each trait.

**Table S6.** Sample sizes and accuracies used for calculating the approximate genetic correlations (weighted Pearson coefficients).

**Table S7.** Spearman correlation between animals in reaction norms regressed on environmental variable and contemporary group effect, for the intercept and slope terms

**Figure S1.** Genetic correlations across environmental gradients using the recommended environmental variable and critical period for each studied trait. The Pearson correlation coefficient (cor) are represented by colors with the mean values (M) shown below. NBD: Number of piglets born dead; MDP: Ultrasound muscle depth (mm) considering an interval of 30 days; and BFT: Ultrasound backfat thickness (mm) considering an interval of 30 days.

**Figure S2.** Spearman correlations among reaction norm terms of the recommended environmental variable and critical period for each studied trait. Letters in the figure means the Spearman correlation between: A) intercept terms; B) intercept and slope; C) slope and intercept; and D) slope terms. Y-axis represents trait 1, and X-axis represents trait 2. TNB: total number of piglets born; NBA: number of piglets born alive; NBD: number of piglets born dead; WN: number of piglets weaned; WW: weaning weight (Kg); IWE: interval between wean to estrus (days); IBF: interval between farrows (days); OTW: off-test weight (Kg); MDP: ultrasound muscle depth (mm); and BFT: ultrasound backfat thickness (mm). For OTW, MDP and BFT considering a critical period of average 30 before measurement date. For WN and WW considering a critical period from 34 days prior to farrowing up to weaning date. For TNB, NBA and NBD considering a critical period of 20 days prior breeding to 30 days into gestation. And for IBF and IWE considering a critical period of 34 days prior farrow to weaning date.

**Table S1.** Description of environmental variables (ENV) for number of piglets born total.

| **ENV^1^** | **Minimum** | **Mean** | **Maximum** | **SD** |
| --- | --- | --- | --- | --- |
| **DewP** | -11.24 | 7.55 | 22.65 | 8.31 |
| **RH** | 16.84 | 63.15 | 98.60 | 13.72 |
| **MeanT** | -7.08 | 14.85 | 28.41 | 7.61 |
| **MaxT** | -3.76 | 21.39 | 37.08 | 7.83 |
| **MinT** | -13.08 | 8.53 | 22.35 | 7.77 |
| **DI** | 8.73 | 25.25 | 35.27 | 5.90 |
| **THI** | 24.26 | 58.70 | 77.39 | 11.09 |

^1^ MaxT: average of maximum daily temperature; MinT: average of minimum daily temperature; MeanT: average of mean daily temperature; DewP: average of daily dew point; RH: average of daily relative humidity; DI: average discomfort index; THI: average temperature-humidity index.

**Table S2.** Accuracies (95% confidence interval) of genomic estimated breeding value for the reaction norm intercept and slope terms considering all environmental variable.

| **Trait^1^** | **Environmental  variable^2^** | **GEBV Accuracies^3^** | | | |
| --- | --- | --- | --- | --- | --- |
|  |  | **Intercept** | | **Slope** | |
|  |  | **Average** | **95% CI** | **Average** | **95% CI** |
| **BFT_30** | **DewP** | 0.7161 | 0.7146 - 0.7176 | 0.4686 | 0.4673 - 0.4700 |
|  | **RH** | 0.7017 | 0.7000 - 0.7033 | 0.5772 | 0.5756 - 0.5787 |
|  | **MeanT** | 0.7085 | 0.7069 - 0.7102 | 0.4443 | 0.4429 - 0.4456 |
|  | **MaxT** | 0.7224 | 0.7209 - 0.7239 | 0.4394 | 0.4380 - 0.4408 |
|  | **MinT** | 0.7029 | 0.7013 - 0.7045 | 0.4198 | 0.4183 - 0.4213 |
|  | **DI** | 0.6976 | 0.6959 - 0.6994 | 0.4118 | 0.4103 - 0.4133 |
|  | **THI** | 0.6960 | 0.6942 - 0.6978 | 0.4135 | 0.4120 - 0.4149 |
| **BFT_120** | **DewP** | 0.7009 | 0.6992 - 0.7026 | 0.4212 | 0.4197 - 0.4227 |
|  | **RH** | 0.6982 | 0.6965 - 0.6999 | 0.4186 | 0.4171 - 0.4201 |
|  | **MeanT** | 0.6855 | 0.6839 - 0.6872 | 0.4153 | 0.4139 - 0.4166 |
|  | **MaxT** | 0.7017 | 0.7000 - 0.7033 | 0.4372 | 0.4356 - 0.4387 |
|  | **MinT** | 0.7151 | 0.7140 - 0.7171 | 0.4677 | 0.4664 - 0.4690 |
|  | **DI** | 0.7107 | 0.7091 - 0.7123 | 0.4440 | 0.4426 - 0.4454 |
|  | **THI** | 0.6940 | 0.6924 - 0.6957 | 0.4506 | 0.4492 - 0.4519 |
| **OTW_30** | **DewP** | 0.6085 | 0.6078 - 0.6098 | 0.5562 | 0.5556 - 0.5567 |
|  | **RH** | 0.5985 | 0.5978 - 0.5990 | 0.5672 | 0.5667 - 0.5675 |
|  | **MeanT** | 0.6456 | 0.6451 - 0.6461 | 0.6022 | 0.6018 - 0.6025 |
|  | **MaxT** | 0.7132 | 0.7128 - 0.7135 | 0.6375 | 0.6371 - 0.6378 |
|  | **MinT** | 0.6419 | 0.6413 - 0.6424 | 0.6503 | 0.6501 - 0.6505 |
|  | **DI** | 0.6277 | 0.6271 - 0.6282 | 0.5928 | 0.5923 - 0.5931 |
|  | **THI** | 0.6275 | 0.6270 - 0.6279 | 0.6347 | 0.6341 - 0.6351 |
| **OTW_120** | **DewP** | 0.6156 | 0.6140 - 0.6171 | 0.4487 | 0.4476 - 0.4498 |
|  | **RH** | 0.6428 | 0.6422 - 0.6433 | 0.4509 | 0.4504 - 0.4517 |
|  | **MeanT** | 0.6461 | 0.6457 - 0.6465 | 0.4682 | 0.4675 - 0.6701 |
|  | **MaxT** | 0.7738 | 0.7720 - 0.7756 | 0.4834 | 0.4791 - 0.4856 |
|  | **MinT** | 0.6319 | 0.6313 - 0.6324 | 0.4279 | 0.4276 - 0.4289 |
|  | **DI** | 0.6405 | 0.6398 - 0.6410 | 0.4608 | 0.4603 - 0.4616 |
|  | **THI** | 0.6658 | 0.6653 - 0.6663 | 0.4909 | 0.4904 - 0.4919 |
| **MDP_30** | **DewP** | 0.6487 | 0.6476 - 0.6494 | 0.3474 | 0.3459 - 0.3489 |
|  | **RH** | 0.6557 | 0.6541 - 0.6574 | 0.3554 | 0.3539 - 0.3569 |
|  | **MeanT** | 0.6629 | 0.6615 - 0.6642 | 0.3377 | 0.3362 - 0.3392 |
|  | **MaxT** | 0.6545 | 0.6529 - 0.6559 | 0.3206 | 0.3192 - 0.3219 |
|  | **MinT** | 0.6488 | 0.6472 - 0.6503 | 0.3181 | 0.3166 - 0.3197 |
|  | **DI** | 0.6532 | 0.6515 - 0.6548 | 0.3103 | 0.3088 - 0.3119 |
|  | **THI** | 0.6405 | 0.6390 - 0.6420 | 0.3173 | 0.3156 - 0.3190 |
| **MDP_120** | **DewP** | 0.6626 | 0.6611 - 0.6640 | 0.3009 | 0.3003 - 0.3018 |
|  | **RH** | 0.6586 | 0.6570 - 0.6601 | 0.3209 | 0.3204 - 0.3214 |
|  | **MeanT** | 0.6515 | 0.6500 - 0.6531 | 0.3082 | 0.3076 - 0.3087 |
|  | **MaxT** | 0.6759 | 0.6745 - 0.6772 | 0.3059 | 0.3047 - 0.3069 |
|  | **MinT** | 0.6606 | 0.6591 - 0.6620 | 0.2547 | 0.2532 - 0.2562 |
|  | **DI** | 0.6518 | 0.6501 - 0.6534 | 0.2842 | 0.2828 - 0.2855 |
|  | **THI** | 0.6360 | 0.6341 - 0.6377 | 0.2633 | 0.2618 - 0.2647 |
| **TNB** | **DewP** | 0.6532 | 0.6526 - 0.6539 | 0.4455 | 0.4449 - 0.4461 |
|  | **RH** | 0.6525 | 0.6519 - 0.6531 | 0.4247 | 0.4241 - 0.4253 |
|  | **MeanT** | 0.6560 | 0.6553 - 0.6566 | 0.4201 | 0.4195 - 0.4208 |
|  | **MaxT** | 0.6562 | 0.6555 - 0.6568 | 0.4702 | 0.4696 - 0.4708 |
|  | **MinT** | 0.6504 | 0.6497 - 0.6510 | 0.4682 | 0.4676 - 0.4688 |
|  | **DI** | 0.6538 | 0.6532 - 0.6544 | 0.4484 | 0.4477 - 0.4490 |
|  | **THI** | 0.6473 | 0.6467 - 0.6479 | 0.4305 | 0.4299 - 0.4312 |
| **NBA** | **DewP** | 0.6384 | 0.6378 - 0.6391 | 0.4564 | 0.4558 - 0.4569 |
|  | **RH** | 0.6411 | 0.6405 - 0.6417 | 0.3809 | 0.3803 - 0.3816 |
|  | **MeanT** | 0.6287 | 0.6281 - 0.6293 | 0.4625 | 0.4619 - 0.4631 |
|  | **MaxT** | 0.6435 | 0.6429 - 0.6441 | 0.4694 | 0.4687 - 0.4700 |
|  | **MinT** | 0.6463 | 0.6450 - 0.6463 | 0.4639 | 0.4633 - 0.4645 |
|  | **DI** | 0.6379 | 0.6372 - 0.6385 | 0.4428 | 0.4422 - 0.4435 |
|  | **THI** | 0.6312 | 0.6306 - 0.6318 | 0.4293 | 0.4286 - 0.4299 |
| **NBD** | **DewP** | 0.6173 | 0.6167 - 0.6180 | 0.4162 | 0.4154 - 0.4168 |
|  | **RH** | 0.6144 | 0.6138 - 0.6150 | 0.4388 | 0.4381 - 0.4394 |
|  | **MeanT** | 0.6239 | 0.6233 - 0.6245 | 0.4131 | 0.4124 - 0.4139 |
|  | **MaxT** | 0.6164 | 0.6157 - 0.6170 | 0.4090 | 0.4082 - 0.4097 |
|  | **MinT** | 0.6189 | 0.6183 - 0.6196 | 0.4201 | 0.4294 - 0.4307 |
|  | **DI** | 0.6165 | 0.6159 - 0.6171 | 0.4266 | 0.4259 - 0.4273 |
|  | **THI** | 0.6128 | 0.6122 - 0.6135 | 0.4206 | 0.4299 - 0.4213 |
| **IBF** | **DewP** | 0.4776 | 0.4769 - 0.4789 | 0.3385 | 0.3377 - 0.3393 |
|  | **RH** | 0.4816 | 0.4809 - 0.4822 | 0.3475 | 0.3467 - 0.3483 |
|  | **MeanT** | 0.4948 | 0.4927 - 0.4940 | 0.3208 | 0.3199 - 0.3217 |
|  | **MaxT** | 0.5313 | 0.5306 - 0.5320 | 0.4587 | 0.4580 - 0.4594 |
|  | **MinT** | 0.5220 | 0.5213 - 0.5226 | 0.4178 | 0.4174 - 0.4181 |
|  | **DI** | 0.5340 | 0.5335 - 0.5344 | 0.4490 | 0.4484 - 0.4494 |
|  | **THI** | 0.5776 | 0.5770 - 0.5782 | 0.4249 | 0.4245 - 0.4252 |
| **IWE** | **DewP** | 0.5453 | 0.5425 - 0.5462 | 0.4216 | 0.4210 - 0.4265 |
|  | **RH** | 0.5523 | 0.5515 - 0.5531 | 0.4026 | 0.4020 - 0.4032 |
|  | **MeanT** | 0.5642 | 0.5635 - 0.5648 | 0.4494 | 0.4490 - 0.4496 |
|  | **MaxT** | 0.6049 | 0.6043 - 0.6054 | 0.4587 | 0.4581 - 0.4594 |
|  | **MinT** | 0.5368 | 0.5361 - 0.5375 | 0.3372 | 0.3365 - 0.3379 |
|  | **DI** | 0.5084 | 0.5075 - 0.5092 | 0.3539 | 0.3535 - 0.3544 |
|  | **THI** | 0.5740 | 0.5733 - 0.5745 | 0.3787 | 0.3780 - 0.3792 |
| **WN_wd** | **DewP** | 0.4367 | 0.4347 - 0.4379 | 0.3165 | 0.3146 - 0.3182 |
|  | **RH** | 0.4680 | 0.4673 - 0.4685 | 0.3693 | 0.3671 - 0.3715 |
|  | **MeanT** | 0.4548 | 0.4542 - 0.4553 | 0.2894 | 0.2883 - 0.2904 |
|  | **MaxT** | 0.3253 | 0.3243 - 0.3262 | 0.3626 | 0.3617 - 0.3634 |
|  | **MinT** | 0.4080 | 0.4075 - 0.4083 | 0.2792 | 0.2781 - 0.2803 |
|  | **DI** | 0.3713 | 0.3704 - 0.3722 | 0.3266 | 0.3255 - 0.3276 |
|  | **THI** | 0.4555 | 0.4546 - 0.4563 | 0.3660 | 0.3657 - 0.3662 |
| **WN_fd** | **DewP** | 0.3245 | 0.3229 - 0.3261 | 0.3178 | 0.3161 - 0.3187 |
|  | **RH** | 0.3121 | 0.4106 - 0.4134 | 0.3475 | 0.3469 - 0.3479 |
|  | **MeanT** | 0.3606 | 0.3588 - 0.3624 | 0.2758 | 0.2751 - 0.2764 |
|  | **MaxT** | 0.4189 | 0.4178 - 0.4198 | 0.2799 | 0.2791 - 0.2806 |
|  | **MinT** | 0.4264 | 0.64254 - 0.4274 | 0.2735 | 0.2730 - 0.2739 |
|  | **DI** | 0.4710 | 0.4701 - 0.4718 | 0.2952 | 0.2942 - 0.2963 |
|  | **THI** | 0.4436 | 0.4432 - 0.4439 | 0.3246 | 0.3227 - 0.3266 |
| **WW_wd** | **DewP** | 0.5091 | 0.5067 - 0.5111 | 0.3976 | 0.3956 - 0.3988 |
|  | **RH** | 0.5206 | 0.5194 - 0.5218 | 0.4726 | 0.4713 - 0.4739 |
|  | **MeanT** | 0.5455 | 0.5452 - 0.5456 | 0.4194 | 0.4190 - 0.4197 |
|  | **MaxT** | 0.4731 | 0.4726 - 0.4736 | 0.3956 | 0.3953 - 0.3958 |
|  | **MinT** | 0.4215 | 0.4208 - 0.4221 | 0.3703 | 0.3700 - 0.3705 |
|  | **DI** | 0.5270 | 0.5266 - 0.5273 | 0.3837 | 0.3832 - 0.3842 |
|  | **THI** | 0.5895 | 0.5892 - 0.5897 | 0.4200 | 0.4196 - 0.4204 |
| **WW_fd** | **DewP** | 0.4687 | 0.4676 - 0.4699 | 0.3756 | 0.3732 - 0.3771 |
|  | **RH** | 0.5172 | 0.5169 - 0.5174 | 0.4126 | 0.4123 - 0.4127 |
|  | **MeanT** | 0.4265 | 0.4261 - 0.4268 | 0.3824 | 0.3821 - 0.3826 |
|  | **MaxT** | 0.4587 | 0.4582 - 0.4592 | 0.3373 | 0.3369 - 0.3376 |
|  | **MinT** | 0.4405 | 0.4401 - 0.4408 | 0.3662 | 0.3657 - 0.3667 |
|  | **DI** | 0.4921 | 0.4917 - 0.4923 | 0.3755 | 0.3747 - 0.3762 |
|  | **THI** | 0.4144 | 0.4139 - 0.4148 | 0.3009 | 0.3006 - 0.3011 |

^1^ OTW_30: off-test weight (Kg) considering an interval of 30 days; OTW_120: off-test weight (Kg) considering an interval of 120 days; MDP_30: ultrasound muscle depth (mm) considering an interval of 30 days; MDP_120: ultrasound muscle depth (mm) considering an interval of 120 days; BFT_30: ultrasound backfat thickness (mm) considering an interval of 30 days; BFT_120: ultrasound backfat thickness (mm) considering an interval of 120 days; TNB: total number of piglets born; NBA: number of piglets born alive; NBD: number of piglets born dead; IWE: interval between wean to estrus (days); IBF: interval between farrows (days); WN_wd: number of piglets weaned considering measurement until weaning date; WN_fd: number of piglets weaned considering measurement until farrow date; WW_wd: weaning weight (Kg) considering measurement until weaning date; WW_fd: weaning weight (Kg) considering measurement until farrow date.

^2^ MaxT: average of maximum daily temperature; MinT: average of minimum daily temperature; MeanT: average of mean daily temperature; DewP: average of daily dew point; RH: average of daily relative humidity; DI: average discomfort index; THI: average temperature-humidity index.

^3^CI: Confidence interval.

**Table S4.** Deviation of genomic estimated breeding value (GEBV) in five different environmental values.

| **Trait^1^** | **Environmental  variable^2^** | **Deviation of GEBV per ENV value^3^** | | | | |
| --- | --- | --- | --- | --- | --- | --- |
|  |  | **First** | **25th** | **50th** | **75th** | **Last** |
| **OTW_30** | **MaxT** | 4.4273 | 3.3972 | 3.3210 | 3.3256 | 3.4815 |
|  | **MinT** | 3.8228 | 3.3953 | 3.3113 | 3.3229 | 3.4419 |
|  | **MeanT** | 4.1777 | 3.3465 | 3.1943 | 3.2196 | 3.4206 |
|  | **DewP** | 2.3412 | 3.0322 | 3.0765 | 3.0525 | 4.0052 |
|  | **RH** | 2.2236 | 3.0534 | 3.3076 | 3.5253 | 4.0518 |
|  | **DI** | 4.1197 | 3.3113 | 3.2805 | 3.4749 | 3.8828 |
|  | **THI** | 4.2502 | 3.3162 | 3.3171 | 3.4834 | 3.8561 |
| **OTW_120** | **MaxT** | 5.5437 | 3.5743 | 3.2391 | 3.1440 | 3.2776 |
|  | **MinT** | 3.2024 | 3.2452 | 3.2686 | 3.2878 | 3.3143 |
|  | **MeanT** | 3.8008 | 3.4927 | 3.3617 | 3.2721 | 3.1411 |
|  | **DewP** | 2.0003 | 3.0060 | 3.5336 | 3.1115 | 3.9299 |
|  | **RH** | 2.0615 | 3.0031 | 3.3324 | 3.6302 | 4.3288 |
|  | **DI** | 2.3649 | 3.0061 | 3.2901 | 3.5274 | 3.8186 |
|  | **THI** | 2.1803 | 2.9316 | 3.2153 | 3.4175 | 3.6856 |
| **MDP_30** | **MaxT** | 1.5645 | 1.7096 | 1.8402 | 1.9567 | 2.1016 |
|  | **MinT** | 1.8513 | 1.8368 | 1.8331 | 1.8313 | 1.8306 |
|  | **MeanT** | 1.5380 | 1.6827 | 1.8371 | 1.9933 | 2.1249 |
|  | **DewP** | 1.6032 | 1.7771 | 1.8982 | 2.0472 | 2.1519 |
|  | **RH** | 1.8241 | 1.9712 | 1.9164 | 2.4730 | 2.5953 |
|  | **DI** | 1.5802 | 1.6772 | 1.7768 | 1.8781 | 1.9503 |
|  | **THI** | 1.4836 | 1.6122 | 1.7315 | 1.8591 | 1.9416 |
| **MDP_120** | **MaxT** | 1.4658 | 1.6109 | 1.7415 | 1.8580 | 2.0029 |
|  | **MinT** | 1.7526 | 1.7381 | 1.7344 | 1.7326 | 1.7319 |
|  | **MeanT** | 1.4393 | 1.5840 | 1.7384 | 1.8946 | 2.0262 |
|  | **DewP** | 1.5045 | 1.6784 | 1.7995 | 1.9485 | 2.0532 |
|  | **RH** | 1.6541 | 1.8361 | 1.9506 | 1.9511 | 2.3253 |
|  | **DI** | 1.4815 | 1.5785 | 1.6781 | 1.7794 | 1.8516 |
|  | **THI** | 1.3849 | 1.5135 | 1.6328 | 1.7604 | 1.8429 |
| **BFT_30** | **MaxT** | 1.7795 | 1.7470 | 1.7095 | 1.6729 | 1.6365 |
|  | **MinT** | 1.6986 | 1.6881 | 1.6790 | 1.6699 | 1.6619 |
|  | **MeanT** | 1.7129 | 1.7017 | 1.6890 | 1.6781 | 1.6690 |
|  | **DewP** | 1.6863 | 1.7045 | 1.7098 | 1.6780 | 1.5410 |
|  | **RH** | 1.8810 | 1.7787 | 1.7146 | 1.6908 | 1.6408 |
|  | **DI** | 1.5565 | 1.5789 | 1.6008 | 1.6226 | 1.6480 |
|  | **THI** | 1.6888 | 1.6657 | 1.6410 | 1.6167 | 1.5912 |
| **BFT_120** | **MaxT** | 1.7648 | 1.7233 | 1.6866 | 1.6532 | 1.6208 |
|  | **MinT** | 1.7481 | 1.7158 | 1.6942 | 1.6753 | 1.6592 |
|  | **MeanT** | 1.7469 | 1.7155 | 1.6801 | 1.6489 | 1.6259 |
|  | **DewP** | 1.6651 | 1.6276 | 1.5989 | 1.5935 | 1.5876 |
|  | **RH** | 1.4975 | 1.5534 | 1.5872 | 1.6170 | 1.6616 |
|  | **DI** | 1.6601 | 1.6597 | 1.6618 | 1.6664 | 1.6737 |
|  | **THI** | 1.6339 | 1.6216 | 1.6177 | 1.6196 | 1.6265 |
| **TNB** | **MaxT** | 0.5256 | 0.5618 | 0.5806 | 0.6434 | 0.6997 |
|  | **MinT** | 0.4999 | 0.5392 | 0.5802 | 0.6320 | 0.6907 |
|  | **MeanT** | 0.4899 | 0.5269 | 0.5749 | 0.6285 | 0.6892 |
|  | **DewP** | 0.4676 | 0.4873 | 0.4987 | 0.5012 | 0.4987 |
|  | **RH** | 0.5137 | 0.5562 | 0.5993 | 0.6432 | 0.6914 |
|  | **DI** | 0.5100 | 0.5423 | 0.5863 | 0.6388 | 0.7051 |
|  | **THI** | 0.5127 | 0.5468 | 0.5824 | 0.6246 | 0.6698 |
| **NBA** | **MaxT** | 0.5405 | 0.4722 | 0.5411 | 0.5572 | 0.6194 |
|  | **MinT** | 0.4459 | 0.4718 | 0.5054 | 0.5531 | 0.6110 |
|  | **MeanT** | 0.4358 | 0.4705 | 0.5134 | 0.5596 | 0.6107 |
|  | **DewP** | 0.4498 | 0.4676 | 0.4987 | 0.5349 | 0.5587 |
|  | **RH** | 0.5028 | 0.5158 | 0.5312 | 0.5484 | 0.5699 |
|  | **DI** | 0.4621 | 0.4884 | 0.5215 | 0.5550 | 0.5982 |
|  | **THI** | 0.4547 | 0.4809 | 0.5166 | 0.5555 | 0.6056 |
| **NBD** | **MaxT** | 0.1188 | 0.1183 | 0.1181 | 0.1178 | 0.1176 |
|  | **MinT** | 0.1141 | 0.1148 | 0.1156 | 0.1164 | 0.1175 |
|  | **MeanT** | 0.1184 | 0.1186 | 0.1188 | 0.1192 | 0.1197 |
|  | **DewP** | 0.0987 | 0.1092 | 0.1087 | 0.1090 | 0.1091 |
|  | **RH** | 0.1185 | 0.1213 | 0.1197 | 0.1192 | 0.1371 |
|  | **DI** | 0.1100 | 0.1130 | 0.1156 | 0.1183 | 0.1213 |
|  | **THI** | 0.1189 | 0.1183 | 0.1180 | 0.1178 | 0.1178 |
| **IBF** | **MaxT** | 1.0771 | 0.9853 | 0.6928 | 0.6274 | 0.6978 |
|  | **MinT** | 0.9599 | 0.6419 | 0.5440 | 0.5348 | 0.5925 |
|  | **MeanT** | 1.2169 | 0.9504 | 0.7052 | 0.5786 | 0.6457 |
|  | **DewP** | 0.9878 | 0.9762 | 0.6762 | 0.6134 | 0.5987 |
|  | **RH** | 1.0181 | 0.8122 | 0.6123 | 0.4257 | 0.2733 |
|  | **DI** | 0.9449 | 0.7343 | 0.6066 | 0.5519 | 0.6111 |
|  | **THI** | 1.1110 | 0.8597 | 0.6492 | 0.5447 | 0.5641 |
| **IWE** | **MaxT** | 0.7373 | 0.5614 | 0.4730 | 0.4256 | 0.5273 |
|  | **MinT** | 0.5153 | 0.4294 | 0.3863 | 0.3700 | 0.3854 |
|  | **MeanT** | 0.3856 | 0.3613 | 0.3747 | 0.4110 | 0.4697 |
|  | **DewP** | 0.4091 | 0.3675 | 0.3610 | 0.3871 | 0.3989 |
|  | **RH** | 0.7132 | 0.3302 | 0.4362 | 0.3512 | 0.1818 |
|  | **DI** | 0.5613 | 0.4534 | 0.3974 | 0.3827 | 0.4146 |
|  | **THI** | 0.5093 | 0.4097 | 0.3938 | 0.4123 | 0.4621 |
| **WN_wd** | **MaxT** | 0.1536 | 0.1448 | 0.2045 | 0.2806 | 0.3284 |
|  | **MinT** | 0.1828 | 0.1825 | 0.2271 | 0.3049 | 0.3790 |
|  | **MeanT** | 0.2192 | 0.1910 | 0.2037 | 0.2494 | 0.3054 |
|  | **DewP** | 0.1982 | 0.1762 | 0.1672 | 0.2198 | 0.2389 |
|  | **RH** | 0.4498 | 0.2322 | 0.2327 | 0.2621 | 0.3850 |
|  | **DI** | 0.2999 | 0.2342 | 0.1784 | 0.1254 | 0.1037 |
|  | **THI** | 0.3889 | 0.2293 | 0.1154 | 0.1461 | 0.2317 |
| **WN_fd** | **MaxT** | 0.0559 | 0.1044 | 0.1650 | 0.2318 | 0.3158 |
|  | **MinT** | 0.1291 | 0.1056 | 0.1410 | 0.2044 | 0.2720 |
|  | **MeanT** | 0.1291 | 0.0806 | 0.1073 | 0.1766 | 0.2657 |
|  | **DewP** | 0.1198 | 0.0982 | 0.1021 | 0.1426 | 0.1892 |
|  | **RH** | 0.4196 | 0.2564 | 0.2286 | 0.1843 | 0.2989 |
|  | **DI** | 0.2107 | 0.1847 | 0.1691 | 0.1640 | 0.1698 |
|  | **THI** | 0.1992 | 0.1677 | 0.1511 | 0.1488 | 0.1586 |
| **WW_wd** | **MaxT** | 0.1110 | 0.1103 | 0.1223 | 0.1404 | 0.1692 |
|  | **MinT** | 0.0976 | 0.1039 | 0.1302 | 0.1718 | 0.2072 |
|  | **MeanT** | 0.1391 | 0.1411 | 0.1472 | 0.1562 | 0.1668 |
|  | **DewP** | 0.1029 | 0.1201 | 0.1299 | 0.1562 | 0.1782 |
|  | **RH** | 0.1810 | 0.0489 | 0.1097 | 0.1807 | 0.2266 |
|  | **DI** | 0.0966 | 0.0960 | 0.0978 | 0.1019 | 0.1065 |
|  | **THI** | 0.0971 | 0.0919 | 0.0978 | 0.1105 | 0.1220 |
| **WW_fd** | **MaxT** | 0.1989 | 0.1734 | 0.1542 | 0.1370 | 0.1164 |
|  | **MinT** | 0.1849 | 0.1590 | 0.1439 | 0.1326 | 0.1279 |
|  | **MeanT** | 0.0825 | 0.0743 | 0.0768 | 0.0870 | 0.1030 |
|  | **DewP** | 0.1092 | 0.1238 | 0.1452 | 0.1246 | 0.1167 |
|  | **RH** | 0.1751 | 0.0991 | 0.1284 | 0.1650 | 0.2194 |
|  | **DI** | 0.0555 | 0.0560 | 0.0633 | 0.0747 | 0.0852 |
|  | **THI** | 0.1173 | 0.1074 | 0.1063 | 0.1123 | 0.1197 |

^1^ OTW_30: off-test weight (Kg) considering an interval of 30 days; OTW_120: off-test weight (Kg) considering an interval of 120 days; MDP_30: ultrasound muscle depth (mm) considering an interval of 30 days; MDP_120: ultrasound muscle depth (mm) considering an interval of 120 days; BFT_30: ultrasound backfat thickness (mm) considering an interval of 30 days; BFT_120: ultrasound backfat thickness (mm) considering an interval of 120 days; TNB: total number of piglets born; NBA: number of piglets born alive; NBD: number of piglets born dead; IWE: interval between wean to estrus (days); IBF: interval between farrows (days); WN_wd: number of piglets weaned considering measurement until weaning date; WN_fd: number of piglets weaned considering measurement until farrow date; WW_wd: weaning weight (Kg) considering measurement until weaning date; WW_fd: weaning weight (Kg) considering measurement until farrow date.

^2^ MaxT: average of maximum daily temperature; MinT: average of minimum daily temperature; MeanT: average of mean daily temperature; DewP: average of daily dew point; RH: average of daily relative humidity; DI: average discomfort index; THI: average temperature-humidity index.

^3^ENV: environmental variable.

**Table S5.** Description of heritability estimates for all analyzed environmental variable for each trait.

| **Trait^1^** | **Environmental variable^2^** | **Heritability^3^** | | | | **σ^2^_a_ Slope** |
| --- | --- | --- | --- | --- | --- | --- |
|  |  | **Min** | **Mean** | **Max** | **SD** |  |
| **OTW_30** | **MaxT** | 0.21 | 0.25 | 0.47 | 0.038 | 52.3510 |
|  | **MeanT** | 0.22 | 0.25 | 0.44 | 0.035 | 39.2530 |
|  | **MinT** | 0.22 | 0.25 | 0.36 | 0.023 | 25.3810 |
|  | **RH** | 0.13 | 0.23 | 0.31 | 0.031 | 3.0420 |
|  | **DI** | 0.23 | 0.28 | 0.46 | 0.045 | 51.8010 |
|  | **THI** | **0.23** | **0.27** | **0.47** | **0.042** | **53.1230** |
|  | **DewP** | 0.16 | 0.23 | 0.3 | 0.035 | 5.8214 |
| **OTW_120** | **MaxT** | **0.22** | **0.24** | **0.39** | **0.029** | **32.8906** |
|  | **MeanT** | 0.20 | 0.22 | 0.23 | 0.007 | 11.9300 |
|  | **MinT** | 0.19 | 0.22 | 0.23 | 0.006 | 11.4001 |
|  | **RH** | 0.12 | 0.24 | 0.32 | 0.037 | 15.5401 |
|  | **DI** | 0.11 | 0.21 | 0.27 | 0.034 | 12.9000 |
|  | **THI** | 0.10 | 0.21 | 0.25 | 0.031 | 13.0000 |
|  | **DewP** | 0.15 | 0.22 | 0.24 | 0.022 | 11.6705 |
| **MDP_30** | **MaxT** | 0.22 | 0.29 | 0.35 | 0.031 | 0.2280 |
|  | **MeanT** | 0.21 | 0.28 | 0.34 | 0.035 | 0.2457 |
|  | **MinT** | 0.22 | 0.28 | 0.32 | 0.028 | 0.2118 |
|  | **RH** | **0.27** | **0.29** | **0.31** | **0.011** | **1.2301** |
|  | **DI** | 0.24 | 0.28 | 0.32 | 0.025 | 0.1110 |
|  | **THI** | 0.20 | 0.25 | 0.30 | 0.028 | 0.1513 |
|  | **DewP** | 0.21 | 0.27 | 0.31 | 0.032 | 0.4489 |
| **MDP_120** | **MaxT** | 0.21 | 0.27 | 0.32 | 0.0315 | 0.1920 |
|  | **MeanT** | 0.22 | 0.26 | 0.29 | 0.019 | 0.2286 |
|  | **MinT** | 0.18 | 0.26 | 0.32 | 0.036 | 0.2016 |
|  | **RH** | **0.23** | **0.30** | **0.35** | **0.028** | **0.9079** |
|  | **DI** | 0.20 | 0.26 | 0.30 | 0.026 | 0.1230 |
|  | **THI** | 0.19 | 0.26 | 0.30 | 0.028 | 0.1256 |
|  | **DewP** | 0.25 | 0.27 | 0.29 | 0.009 | 0.3949 |
| **BFT_30** | **MaxT** | 0.41 | 0.42 | 0.44 | 0.005 | 0.375 |
|  | **MeanT** | 0.41 | 0.42 | 0.43 | 0.006 | 0.197 |
|  | **MinT** | 0.41 | 0.42 | 0.42 | 0.001 | 0.234 |
|  | **RH** | **0.38** | **0.42** | **0.47** | **0.019** | **1.025** |
|  | **DI** | 0.39 | 0.41 | 0.42 | 0.005 | 0.145 |
|  | **THI** | 0.39 | 0.40 | 0.41 | 0.002 | 0.102 |
|  | **DewP** | 0.4 | 0.41 | 0.42 | 0.002 | 0.366 |
| **BFT_120** | **MaxT** | 0.41 | 0.43 | 0.46 | 0.012 | 0.6488 |
|  | **MeanT** | 0.41 | 0.42 | 0.44 | 0.009 | 0.3333 |
|  | **MinT** | 0.42 | 0.44 | 0.47 | 0.143 | 0.6495 |
|  | **RH** | **0.40** | **0.41** | **0.43** | **0.005** | **0.9645** |
|  | **DI** | 0.41 | 0.42 | 0.44 | 0.007 | 0.6815 |
|  | **THI** | 0.41 | 0.41 | 0.42 | 0.003 | 0.7001 |
|  | **DewP** | 0.4 | 0.41 | 0.41 | 0.0008 | 0.3320 |
| **TNB** | **MaxT** | **0.09** | **0.11** | **0.12** | **0.008** | **0.2095** |
|  | **MeanT** | 0.08 | 0.10 | 0.13 | 0.012 | 0.1566 |
|  | **MinT** | 0.08 | 0.10 | 0.13 | 0.015 | 0.2014 |
|  | **RH** | 0.09 | 0.10 | 0.12 | 0.006 | 0.1042 |
|  | **DI** | 0.09 | 0.10 | 0.13 | 0.01 | 0.1820 |
|  | **THI** | 0.09 | 0.10 | 0.12 | 0.007 | 0.2013 |
|  | **DewP** | 0.09 | 0.11 | 0.13 | 0.011 | 0.1584 |
| **NBA** | **MaxT** | **0.08** | **0.09** | **0.12** | **0.011** | **0.2727** |
|  | **MeanT** | 0.08 | 0.09 | 0.12 | 0.012 | 0.2160 |
|  | **MinT** | 0.08 | 0.09 | 0.12 | 0.012 | 0.2086 |
|  | **RH** | 0.08 | 0.09 | 0.10 | 0.002 | 0.1072 |
|  | **DI** | 0.08 | 0.09 | 0.11 | 0.01 | 0.0364 |
|  | **THI** | 0.08 | 0.09 | 0.11 | 0.007 | 0.1998 |
|  | **DewP** | 0.08 | 0.09 | 0.12 | 0.011 | 0.1663 |
| **NBD** | **MaxT** | 0.05 | 0.06 | 0.06 | 0.0025 | 0.0022 |
|  | **MeanT** | 0.06 | 0.06 | 0.07 | 0.001 | 0.0039 |
|  | **MinT** | 0.05 | 0.06 | 0.06 | 0.002 | 0.0040 |
|  | **RH** | **0.04** | **0.06** | **0.09** | **0.009** | **0.0122** |
|  | **DI** | 0.05 | 0.06 | 0.06 | 0.003 | 0.0030 |
|  | **THI** | 0.05 | 0.06 | 0.06 | 0.003 | 0.0032 |
|  | **DewP** | 0.05 | 0.06 | 0.06 | 0.003 | 0.0029 |
| **IBF** | **MaxT** | **0.03** | **0.04** | **0.1** | **0.017** | **4.5055** |
|  | **MeanT** | 0.03 | 0.04 | 0.09 | 0.015 | 4.1785 |
|  | **MinT** | 0.02 | 0.04 | 0.08 | 0.015 | 3.4433 |
|  | **RH** | 0.02 | 0.03 | 0.06 | 0.011 | 1.6632 |
|  | **DI** | 0.03 | 0.05 | 0.11 | 0.022 | 3.4949 |
|  | **THI** | 0.03 | 0.05 | 0.12 | 0.022 | 4.2014 |
|  | **DewP** | 0.02 | 0.03 | 0.07 | 0.012 | 2.7972 |
| **IWE** | **MaxT** | **0.04** | **0.05** | **0.08** | **0.011** | **0.9816** |
|  | **MeanT** | 0.04 | 0.05 | 0.07 | 0.008 | 0.9647 |
|  | **MinT** | 0.04 | 0.05 | 0.08 | 0.01 | 0.7099 |
|  | **RH** | 0.01 | 0.05 | 0.11 | 0.022 | 0.4463 |
|  | **DI** | 0.04 | 0.05 | 0.09 | 0.013 | 0.8346 |
|  | **THI** | 0.04 | 0.05 | 0.09 | 0.011 | 0.8709 |
|  | **DewP** | 0.04 | 0.05 | 0.07 | 0.008 | 0.5505 |
| **WN_wd** | **MaxT** | 0.03 | 0.06 | 0.14 | 0.028 | 0.4102 |
|  | **MeanT** | 0.04 | 0.06 | 0.12 | 0.021 | 0.5012 |
|  | **MinT** | 0.01 | 0.07 | 0.12 | 0.014 | 0.4107 |
|  | **RH** | **0.07** | **0.11** | **0.31** | **0.048** | **1.4734** |
|  | **DI** | 0.06 | 0.08 | 0.15 | 0.023 | 0.6017 |
|  | **THI** | 0.06 | 0.09 | 0.16 | 0.025 | 0.7874 |
|  | **DewP** | 0.06 | 0.1 | 0.22 | 0.038 | 0.9881 |
| **WN_fd** | **MaxT** | 0.07 | 0.11 | 0.22 | 0.032 | 1.0787 |
|  | **MeanT** | 0.06 | 0.09 | 0.18 | 0.029 | 0.9576 |
|  | **MinT** | 0.08 | 0.11 | 0.19 | 0.027 | 0.9416 |
|  | **RH** | **0.09** | **0.14** | **0.36** | **0.048** | **1.2910** |
|  | **DI** | 0.06 | 0.10 | 0.21 | 0.037 | 0.7189 |
|  | **THI** | 0.09 | 0.14 | 0.26 | 0.038 | 1.0633 |
|  | **DewP** | 0.03 | 0.09 | 0.029 | 0.042 | 0.9527 |
| **WW_wd** | **MaxT** | 0.05 | 0.07 | 0.1 | 0.009 | 0.0460 |
|  | **MeanT** | 0.05 | 0.06 | 0.07 | 0.004 | 0.1010 |
|  | **MinT** | 0.06 | 0.07 | 0.13 | 0.015 | 0.1380 |
|  | **RH** | **0.05** | **0.08** | **0.26** | **0.036** | **0.7150** |
|  | **DI** | 0.05 | 0.07 | 0.10 | 0.011 | 0.0960 |
|  | **THI** | 0.07 | 0.08 | 0.12 | 0.014 | 0.1230 |
|  | **DewP** | 0.06 | 0.08 | 0.12 | 0.012 | 0.1733 |
| **WW_fd** | **MaxT** | 0.05 | 0.06 | 0.11 | 0.012 | 0.1956 |
|  | **MeanT** | 0.07 | 0.09 | 0.12 | 0.011 | 0.1674 |
|  | **MinT** | 0.06 | 0.08 | 0.11 | 0.013 | 0.1705 |
|  | **RH** | **0.01** | **0.05** | **0.14** | **0.035** | **0.3734** |
|  | **DI** | 0.06 | 0.07 | 0.07 | 0.005 | 0.0770 |
|  | **THI** | 0.05 | 0.06 | 0.10 | 0.012 | 0.1742 |
|  | **DewP** | 0.01 | 0.03 | 0.08 | 0.025 | 0.1013 |

Cases highlighted in bold show the strongest estimate of the slope term variance, per trait

^1^ OTW_30: off-test weight (Kg) considering an interval of 30 days; OTW_120: off-test weight (Kg) considering an interval of 120 days; MDP_30: ultrasound muscle depth (mm) considering an interval of 30 days; MDP_120: ultrasound muscle depth (mm) considering an interval of 120 days; BFT_30: ultrasound backfat thickness (mm) considering an interval of 30 days; BFT_120: ultrasound backfat thickness (mm) considering an interval of 120 days; TNB: total number of piglets born; NBA: number of piglets born alive; NBD: number of piglets born dead; IWE: interval between wean to estrus (days); IBF: interval between farrows (days); WN_wd: number of piglets weaned considering measurement until weaning date; WN_fd: number of piglets weaned considering measurement until farrow date; WW_wd: weaning weight (Kg) considering measurement until weaning date; WW_fd: weaning weight (Kg) considering measurement until farrow date.

^2^ MaxT: average of maximum daily temperature; MinT: average of minimum daily temperature; MeanT: average of mean daily temperature; DewP: average of daily dew point; RH: average of daily relative humidity; DI: average discomfort index; THI: average temperature-humidity index

^3^ Min: minimum; Max: maximum; SD: standard deviation.

**Table S6.** Sample sizes and accuracies used for calculating the approximate genetic correlations (weighted Pearson coefficients).

| **Trait 1^1^** | **Trait 2** | **Number of animals** | **Average accuracies of GEBV (95% CI)^2^** | |
| --- | --- | --- | --- | --- |
|  |  |  | **Trait 1** | **Trait 2** |
| **NBA_int** | **TNB_int** | 84,270 | 0.6618 (0.6613 - 0.6623) | 0.6756 (0.6751 - 0.6761) |
| **NBA_int** | **TNB_slope** | 84,270 | 0.6618 (0.6613 - 0.6623) | 0.4543 (0.4538 - 0.4547) |
| **NBA_int** | **NBD_int** | 82,485 | 0.6624 (0.6618 - 0.6628) | 0.4648 (0.4642 - 0.4653) |
| **NBA_int** | **NBD_slope** | 82,485 | 0.6624 (0.6618 - 0.6628) | 0.4648 (0.4642 - 0.4653) |
| **NBA_int** | **WN_int** | 8,472 | 0.7552 (0.7532 - 0.7572) | 0.4938 (0.4919 - 0.4957) |
| **NBA_int** | **WN_slope** | 8,472 | 0.7552 (0.7532 - 0.7572) | 0.4407 (0.4390 - 0.4424) |
| **NBA_int** | **WW_int** | 8,118 | 0.7608 (0.7589 - 0.7627) | 0.4733 (0.4713 - 0.4751) |
| **NBA_int** | **WW_slope** | 8,118 | 0.7608 (0.7589 - 0.7627) | 0.4571 (0.4550 - 0.4590) |
| **NBA_int** | **IWE_int** | 71,564 | 0.6673 (0.6667 - 0.6678) | 0.5859 (0.5854 - 0.5864) |
| **NBA_int** | **IWE_slope** | 71,564 | 0.6673 (0.6667 - 0.6678) | 0.4807 (0.4801 - 0.4812) |
| **NBA_int** | **IBF_int** | 64,635 | 0.6750 (0.6744 - 0.6756) | 0.5532 (0.5525 - 0.5537) |
| **NBA_int** | **IBF_slope** | 64,635 | 0.6750 (0.6744 - 0.6756) | 0.4817 (0.4810 - 0.4822) |
| **NBA_int** | **OTW_int** | 48,733 | 0.6817 (0.6809 - 0.6823) | 0.6302 (0.6293 - 0.6310( |
| **NBA_int** | **OTW_slope** | 48,733 | 0.6817 (0.6809 - 0.6823) | 0.5414 (0.5406 - 0.5422) |
| **NBA_int** | **MDP_int** | 8,087 | 0.7551 (0.7530 - 0.7571) | 0.6578 (0.6551 - 0.6604) |
| **NBA_int** | **MDP_slope** | 8,087 | 0.7551 (0.7530 - 0.7571) | 0.4306 (0.4288 - 0.4324) |
| **NBA_int** | **BFT_int** | 8,425 | 0.7585 (0.7566 - 0.7604) | 0.7047 (0.7021 - 0.7072) |
| **NBA_int** | **BFT_slope** | 8,425 | 0.7585 (0.7566 - 0.7604) | 0.4443 (0.4424 - 0.4461) |
| **NBA_slope** | **TNB_int** | 84,270 | 0.4627 (0.4621 - 0.4631) | 0.6756 (0.6751 - 0.6761) |
| **NBA_slope** | **TNB_slope** | 84,270 | 0.4627 (0.4621 - 0.4631) | 0.4543 (0.4538 - 0.4547) |
| **NBA_slope** | **NBD_int** | 82,485 | 0.4623 (0.4617 - 0.4627 | 0.6341 (0.6335 - 0.6345) |
| **NBA_slope** | **NBD_slope** | 82,485 | 0.4623 (0.4617 - 0.4627 | 0.4648 (0.4642 - 0.4653) |
| **NBA_slope** | **WN_int** | 8,472 | 0.5313 (0.5292 - 0.5333) | 0.4938 (0.4919 - 0.4957) |
| **NBA_slope** | **WN_slope** | 8,472 | 0.5313 (0.5292 - 0.5333) | 0.4407 (0.4390 - 0.4424) |
| **NBA_slope** | **WW_int** | 8,118 | 0.5346 (0.5326 - 0.5366) | 0.4733 (0.4713 - 0.4751) |
| **NBA_slope** | **WW_slope** | 8,118 | 0.5346 (0.5326 - 0.5366) | 0.4571 (0.4550 - 0.4590) |
| **NBA_slope** | **IWE_int** | 71,564 | 0.4652 (0.4646 - 0.4657) | 0.5859 (0.5854 - 0.5864) |
| **NBA_slope** | **IWE_slope** | 71,564 | 0.4652 (0.4646 - 0.4657) | 0.4807 (0.4801 - 0.4812) |
| **NBA_slope** | **IBF_int** | 64,635 | 0.4682 (0.4676 - 0.4688) | 0.5532 (0.5525 - 0.5537) |
| **NBA_slope** | **IBF_slope** | 64,635 | 0.4682 (0.4676 - 0.4688) | 0.4817 (0.4810 - 0.4822) |
| **NBA_slope** | **OTW_int** | 48,733 | 0.4728 (0.4721 - 0.4735) | 0.6302 (0.6293 - 0.6310( |
| **NBA_slope** | **OTW_slope** | 48,733 | 0.4728 (0.4721 - 0.4735) | 0.5414 (0.5406 - 0.5422) |
| **NBA_slope** | **MDP_int** | 8,087 | 0.5305 (0.5284 - 0.5325) | 0.6578 (0.6551 - 0.6604) |
| **NBA_slope** | **MDP_slope** | 8,087 | 0.5305 (0.5284 - 0.5325) | 0.4306 (0.4288 - 0.4324) |
| **NBA_slope** | **BFT_int** | 8,425 | 0.5340 (0.5320 - 0.5360) | 0.7047 (0.7021 - 0.7072) |
| **NBA_slope** | **BFT_slope** | 8,425 | 0.5340 (0.5320 - 0.5360) | 0.4443 (0.4424 - 0.4461) |
| **TNB_int** | **NBD_int** | 81,856 | 0.6763 (0.6758 - 0.6768) | 0.6342 (0.6337 - 0.6347) |
| **TNB_int** | **NBD_slope** | 81,856 | 0.6763 (0.6758 - 0.6768) | 0.4650 (0.4644 - 0.4654) |
| **TNB_int** | **WN_int** | 8,207 | 0.7677 (0.7656 - 0.7697) | 0.4945 (0.4925 - 0.4964) |
| **TNB_int** | **WN_slope** | 8,207 | 0.7677 (0.7656 - 0.7697) | 0.4408 (0.4390 - 0.4425) |
| **TNB_int** | **WW_int** | 7,790 | 0.7740 (0.7720 - 0.7759) | 0.4736 (0.4715 - 0.4755) |
| **TNB_int** | **WW_slope** | 7,790 | 0.7740 (0.7720 - 0.7759) | 0.4572 (0.4551 - 0.4592) |
| **TNB_int** | **IWE_int** | 70,845 | 0.6815 (0.6809 - 0.6821) | 0.5862 (0.5857 - 0.5867) |
| **TNB_int** | **IWE_slope** | 70,845 | 0.6815 (0.6809 - 0.6821) | 0.4809 (0.4803 - 0.4814) |
| **TNB_int** | **IBF_int** | 64,057 | 0.6893 (0.6886 - 0.6898) | 0.5538 (0.5531 - 0.5543) |
| **TNB_int** | **IBF_slope** | 64,057 | 0.6893 (0.6886 - 0.6898) | 0.4819 (0.4813 - 0.4824) |
| **TNB_int** | **OTW_int** | 48,514 | 0.6947 (0.6940 - 0.6954) | 0.6294 (0.6285 - 0.6302) |
| **TNB_int** | **OTW_slope** | 48,514 | 0.6947 (0.6940 - 0.6954) | 0.5402 (0.5393 - 0.5409) |
| **TNB_int** | **MDP_int** | 7,824 | 0.7673 (0.7652 - 0.7694) | 0.6574 (0.6547 - 0.6601) |
| **TNB_int** | **MDP_slope** | 7,824 | 0.7673 (0.7652 - 0.7694) | 0.4311 (0.4292 - 0.4328) |
| **TNB_int** | **BFT_int** | 8,135 | 0.7713 (0.7692 - 0.7732) | 0.7043 (0.7017 - 0.7069) |
| **TNB_int** | **BFT_slope** | 8,135 | 0.7713 (0.7692 - 0.7732) | 0.4444 (0.4424 - 0.4462) |
| **TNB_slope** | **NBD_int** | 81,856 | 0.4538 (0.4533 - 0.4543) | 0.6342 (0.6337 - 0.6347) |
| **TNB_slope** | **NBD_slope** | 81,856 | 0.4538 (0.4533 - 0.4543) | 0.4650 (0.4644 - 0.4654) |
| **TNB_slope** | **WN_int** | 8,207 | 0.5126 (0.5105 - 0.5146) | 0.4945 (0.4925 - 0.4964) |
| **TNB_slope** | **WN_slope** | 8,207 | 0.5126 (0.5105 - 0.5146) | 0.4408 (0.4390 - 0.4425) |
| **TNB_slope** | **WW_int** | 7,790 | 0.5159 (0.5138 - 0.5179) | 0.4736 (0.4715 - 0.4755) |
| **TNB_slope** | **WW_slope** | 7,790 | 0.5159 (0.5138 - 0.5179) | 0.4572 (0.4551 - 0.4592) |
| **TNB_slope** | **IWE_int** | 70,845 | 0.4561 (0.4555 - 0.4566) | 0.5862 (0.5857 - 0.5867) |
| **TNB_slope** | **IWE_slope** | 70,845 | 0.4561 (0.4555 - 0.4566) | 0.4809 (0.4803 - 0.4814) |
| **TNB_slope** | **IBF_int** | 64,057 | 0.4589 (0.4583 - 0.4595) | 0.5538 (0.5531 - 0.5543) |
| **TNB_slope** | **IBF_slope** | 64,057 | 0.4589 (0.4583 - 0.4595) | 0.4819 (0.4813 - 0.4824) |
| **TNB_slope** | **OTW_int** | 48,514 | 0.4628 (0.4621 - 0.4635) | 0.6294 (0.6285 - 0.6302) |
| **TNB_slope** | **OTW_slope** | 48,514 | 0.4628 (0.4621 - 0.4635) | 0.5402 (0.5393 - 0.5409) |
| **TNB_slope** | **MDP_int** | 7,824 | 0.5134 (0.5113 - 0.5155) | 0.6574 (0.6547 - 0.6601) |
| **TNB_slope** | **MDP_slope** | 7,824 | 0.5134 (0.5113 - 0.5155) | 0.4311 (0.4292 - 0.4328) |
| **TNB_slope** | **BFT_int** | 8,135 | 0.5152 (0.5131 - 0.5171) | 0.7043 (0.7017 - 0.7069) |
| **TNB_slope** | **BFT_slope** | 8,135 | 0.5152 (0.5131 - 0.5171) | 0.4444 (0.4424 - 0.4462) |
| **NBD_int** | **WN_int** | 8,189 | 0.7239 (0.7218 - 0.7258) | 0.4941 (0.4921 - 0.4960) |
| **NBD_int** | **WN_slope** | 8,189 | 0.7239 (0.7218 - 0.7258) | 0.4410 (0.4392 - 0.4427) |
| **NBD_int** | **WW_int** | 7,848 | 0.7292 (0.7272 - 0.7311) | 0.4734 (0.4714 - 0.4753) |
| **NBD_int** | **WW_slope** | 7,848 | 0.7292 (0.7272 - 0.7311) | 0.4572 (0.4551 - 0.4591_ |
| **NBD_int** | **IWE_int** | 69,977 | 0.6395 (0.6389 - 0.6400) | 0.5864 (0.5858 - 0.5869) |
| **NBD_int** | **IWE_slope** | 69,977 | 0.6395 (0.6389 - 0.6400) | 0.4799 (0.4793 - 0.4804) |
| **NBD_int** | **IBF_int** | 63,190 | 0.6461 (0.6455 - 0.6466) | 0.5538 (0.5532 - 0.5543) |
| **NBD_int** | **IBF_slope** | 63,190 | 0.6461 (0.6455 - 0.6466) | 0.4809 (0.4803 - 0.4815) |
| **NBD_int** | **OTW_int** | 47,282 | 0.6536 (0.6529 - 0.6543) | 0.6294 (0.6286 - 0.6302) |
| **NBD_int** | **OTW_slope** | 47,282 | 0.6536 (0.6529 - 0.6543) | 0.5385 (0.5376 - 0.5393) |
| **NBD_int** | **MDP_int** | 7,786 | 0.7240 (0.7219 - 0.7260) | 0.6572 (0.6545 - 0.6599) |
| **NBD_int** | **MDP_slope** | 7,786 | 0.7240 (0.7219 - 0.7260) | 0.4307 (0.4288 - 0.4324) |
| **NBD_int** | **BFT_int** | 8,097 | 0.7280 (0.7259 - 0.7299) | 0.7050 (0.7023 - 0.7075) |
| **NBD_int** | **BFT_slope** | 8,097 | 0.7280 (0.7259 - 0.7299) | 0.4451 (0.4432 - 0.4479) |
| **NBD_slope** | **WN_int** | 8,189 | 0.5177 (0.5156 - 0.5197) | 0.4941 (0.4921 - 0.4960) |
| **NBD_slope** | **WN_slope** | 8,189 | 0.5177 (0.5156 - 0.5197) | 0.4410 (0.4392 - 0.4427) |
| **NBD_slope** | **WW_int** | 7,848 | 0.5208 (0.5186 - 0.5228) | 0.4734 (0.4714 - 0.4753) |
| **NBD_slope** | **WW_slope** | 7,848 | 0.5208 (0.5186 - 0.5228) | 0.4572 (0.4551 - 0.4591_ |
| **NBD_slope** | **IWE_int** | 69,977 | 0.4675 (0.4669 - 0.4680) | 0.5864 (0.5858 - 0.5869) |
| **NBD_slope** | **IWE_slope** | 69,977 | 0.4675 (0.4669 - 0.4680) | 0.4799 (0.4793 - 0.4804) |
| **NBD_slope** | **IBF_int** | 63,190 | 0.4705 (0.4699 - 0.4711) | 0.5538 (0.5532 - 0.5543) |
| **NBD_slope** | **IBF_slope** | 63,190 | 0.4705 (0.4699 - 0.4711) | 0.4809 (0.4803 - 0.4815) |
| **NBD_slope** | **OTW_int** | 47,282 | 0.4775 (0.4768 - 0.4782) | 0.6294 (0.6286 - 0.6302) |
| **NBD_slope** | **OTW_slope** | 47,282 | 0.4775 (0.4768 - 0.4782) | 0.5385 (0.5376 - 0.5393) |
| **NBD_slope** | **MDP_int** | 7,786 | 0.5166 (0.5145 - 0.5187) | 0.6572 (0.6545 - 0.6599) |
| **NBD_slope** | **MDP_slope** | 7,786 | 0.5166 (0.5145 - 0.5187) | 0.4307 (0.4288 - 0.4324) |
| **NBD_slope** | **BFT_int** | 8,097 | 0.5198 (0.5176 - 0.5218) | 0.7050 (0.7023 - 0.7075) |
| **NBD_slope** | **BFT_slope** | 8,097 | 0.5198 (0.5176 - 0.5218) | 0.4451 (0.4432 - 0.4479) |
| **WN_int** | **WW_int** | 5,873 | 0.5062 (0.5039 _ 0.5084) | 0.4790 (0.4766 - 0.4813) |
| **WN_int** | **WW_slope** | 5,873 | 0.5062 (0.5039 _ 0.5084) | 0.4640 (0.4615 - 0.4663) |
| **WN_int** | **IWE_int** | 8,503 | 0.4933 (0.4913 - 0.4951) | 0.6569 (0.6550 - 0.6587) |
| **WN_int** | **IWE_slope** | 8,503 | 0.4933 (0.4913 - 0.4951) | 0.5340 (0.5320 - 0.5360) |
| **WN_int** | **IBF_int** | 8,525 | 0.4938 (0.4919 - 0.4957) | 0.6231 (0.6212 - 0.6250) |
| **WN_int** | **IBF_slope** | 8,525 | 0.4938 (0.4919 - 0.4957) | 0.5387 (0.5367 - 0.5406) |
| **WN_int** | **OTW_int** | 8,700 | 0.4928 (0.4908 - 0.4946) | 0.7035 (0.7010 - 0.7058) |
| **WN_int** | **OTW_slope** | 8,700 | 0.4928 (0.4908 - 0.4946) | 0.6144 (0.6121 - 0.6165) |
| **WN_int** | **MDP_int** | 5,718 | 0.4995 (0.4972 - 0.5017) | 0.6731 (0.6699 - 0.6761) |
| **WN_int** | **MDP_slope** | 5,718 | 0.4995 (0.4972 - 0.5017) | 0.4362 (0.4340 - 0.4382) |
| **WN_int** | **BFT_int** | 5,917 | 0.5049 (0.5026 - 0.5070) | 0.7216 (0.7187 - 0.7244) |
| **WN_int** | **BFT_slope** | 5,917 | 0.5049 (0.5026 - 0.5070) | 0.4521 (0.4498 - 0.4543) |
| **WN_slope** | **WW_int** | 5,873 | 0.4487 (0.4465 - 0.4507) | 0.4790 (0.4766 - 0.4813) |
| **WN_slope** | **WW_slope** | 5,873 | 0.4487 (0.4465 - 0.4507) | 0.4640 (0.4615 - 0.4663) |
| **WN_slope** | **IWE_int** | 8,503 | 0.4404 (0.4386 - 0.4421) | 0.6569 (0.6550 - 0.6587) |
| **WN_slope** | **IWE_slope** | 8,503 | 0.4404 (0.4386 - 0.4421) | 0.5340 (0.5320 - 0.5360) |
| **WN_slope** | **IBF_int** | 8,525 | 0.4406 (0.4388 - 0.4423) | 0.6231 (0.6212 - 0.6250) |
| **WN_slope** | **IBF_slope** | 8,525 | 0.4406 (0.4388 - 0.4423) | 0.5387 (0.5367 - 0.5406) |
| **WN_slope** | **OTW_int** | 8,700 | 0.4398 (0.4381 - 0.4415) | 0.7035 (0.7010 - 0.7058) |
| **WN_slope** | **OTW_slope** | 8,700 | 0.4398 (0.4381 - 0.4415) | 0.6144 (0.6121 - 0.6165) |
| **WN_slope** | **MDP_int** | 5,718 | 0.4451 (0.4429 - 0.4472) | 0.6731 (0.6699 - 0.6761) |
| **WN_slope** | **MDP_slope** | 5,718 | 0.4451 (0.4429 - 0.4472) | 0.4362 (0.4340 - 0.4382) |
| **WN_slope** | **BFT_int** | 5,917 | 0.4482 (0.4460 - 0.4503) | 0.7216 (0.7187 - 0.7244) |
| **WN_slope** | **BFT_slope** | 5,917 | 0.4482 (0.4460 - 0.4503) | 0.4521 (0.4498 - 0.4543) |
| **WW_int** | **IWE_int** | 8,109 | 0.4727 (0.4707 - 0.4746) | 0.6616 (0.6598 - 0.6634) |
| **WW_int** | **IWE_slope** | 8,109 | 0.4727 (0.4707 - 0.4746) | 0.5369 (0.5348 - 0.5389) |
| **WW_int** | **IBF_int** | 8,167 | 0.4732 (0.4712 - 0.4750) | 0.6265 (0.6245 - 0.6283) |
| **WW_int** | **IBF_slope** | 8,167 | 0.4732 (0.4712 - 0.4750) | 0.5429 (0.5409 - 0.5448) |
| **WW_int** | **OTW_int** | 16,981 | 0.4652 (0.4640 - 0.4664) | 0.6914 (0.6902 - 0.6926) |
| **WW_int** | **OTW_slope** | 16,981 | 0.4652 (0.4640 - 0.4664) | 0.6018 (0.6005 - 0.6030) |
| **WW_int** | **MDP_int** | 11,127 | 0.4702 (0.4687 - 0.4717) | 0.6963 (0.6947 - 0.6979) |
| **WW_int** | **MDP_slope** | 11,127 | 0.4702 (0.4687 - 0.4717) | 0.4242 (0.4227 - 0.4255) |
| **WW_int** | **BFT_int** | 12,373 | 0.4705 (0.4690 - 0.4718) | 0.7458 (0.7446 - 0.7472) |
| **WW_int** | **BFT_slope** | 12,373 | 0.4705 (0.4690 - 0.4718) | 0.4443 (0.4428 - 0.4456) |
| **WW_slope** | **IWE_int** | 8,109 | 0.4568 (0.4548 - 0.4588) | 0.6616 (0.6598 - 0.6634) |
| **WW_slope** | **IWE_slope** | 8,109 | 0.4568 (0.4548 - 0.4588) | 0.5369 (0.5348 - 0.5389) |
| **WW_slope** | **IBF_int** | 8,167 | 0.4568 (0.4548 - 0.4587) | 0.6265 (0.6245 - 0.6283) |
| **WW_slope** | **IBF_slope** | 8,167 | 0.4568 (0.4548 - 0.4587) | 0.5429 (0.5409 - 0.5448) |
| **WW_slope** | **OTW_int** | 16,981 | 0.4504 (0.4491 - 0.4515) | 0.6914 (0.6902 - 0.6926) |
| **WW_slope** | **OTW_slope** | 16,981 | 0.4504 (0.4491 - 0.4515) | 0.6018 (0.6005 - 0.6030) |
| **WW_slope** | **MDP_int** | 11,127 | 0.4534 (0.4518 - 0.4549) | 0.6963 (0.6947 - 0.6979) |
| **WW_slope** | **MDP_slope** | 11,127 | 0.4534 (0.4518 - 0.4549) | 0.4242 (0.4227 - 0.4255) |
| **WW_slope** | **BFT_int** | 12,373 | 0.4571 (0.4556 - 0.4585) | 0.7458 (0.7446 - 0.7472) |
| **WW_slope** | **BFT_slope** | 12,373 | 0.4571 (0.4556 - 0.4585) | 0.4443 (0.4428 - 0.4456) |
| **IWE_int** | **IBF_int** | 62,648 | 0.5913 (0.5907 - 0.5918) | 0.5534 (0.5527 - 0.5539) |
| **IWE_int** | **IBF_slope** | 62,648 | 0.5913 (0.5907 - 0.5918) | 0.4816 (0.4809 - 0.4821) |
| **IWE_int** | **OTW_int** | 44,767 | 0.6012 (0.0.6005 - 0.6019) | 0.6277 (0.6268 - 0.6286) |
| **IWE_int** | **OTW_slope** | 44,767 | 0.6012 (0.0.6005 - 0.6019) | 0.5403 (0.5393 - 0.5411) |
| **IWE_int** | **MDP_int** | 8,105 | 0.6573 (0.6554 - 0.6591) | 0.6559 (0.6532 - 0.6585) |
| **IWE_int** | **MDP_slope** | 8,105 | 0.6573 (0.6554 - 0.6591) | 0.4302 (0.4283 - 0.4319) |
| **IWE_int** | **BFT_int** | 8,434 | 0.6608 (0.6589 - 0.6625) | 0.7033 (0.7007 - 0.7058) |
| **IWE_int** | **BFT_slope** | 8,434 | 0.6608 (0.6589 - 0.6625) | 0.4440 (0.4421 - 0.4458) |
| **IWE_slope** | **IBF_int** | 62,648 | 0.4835 (0.4829 - 0.4841) | 0.5534 (0.5527 - 0.5539) |
| **IWE_slope** | **IBF_slope** | 62,648 | 0.4835 (0.4829 - 0.4841) | 0.4816 (0.4809 - 0.4821) |
| **IWE_slope** | **OTW_int** | 44,767 | 0.4882 (0.4874 - 0.4889) | 0.6277 (0.6268 - 0.6286) |
| **IWE_slope** | **OTW_slope** | 44,767 | 0.4882 (0.4874 - 0.4889) | 0.5403 (0.5393 - 0.5411) |
| **IWE_slope** | **MDP_int** | 8,105 | 0.5351 (0.5333 - 0.5371) | 0.6559 (0.6532 - 0.6585) |
| **IWE_slope** | **MDP_slope** | 8,105 | 0.5351 (0.5333 - 0.5371) | 0.4302 (0.4283 - 0.4319) |
| **IWE_slope** | **BFT_int** | 8,434 | 0.5372 (0.5352 - 0.5391) | 0.7033 (0.7007 - 0.7058) |
| **IWE_slope** | **BFT_slope** | 8,434 | 0.5372 (0.5352 - 0.5391) | 0.4440 (0.4421 - 0.4458) |
| **IBF_int** | **OTW_int** | 41,380 | 0.5667 (0.5659 - 0.5674) | 0.6274 (0.6263 - 0.6283) |
| **IBF_int** | **OTW_slope** | 41,380 | 0.5667 (0.5659 - 0.5674) | 0.5429 (0.5419 - 0.5438) |
| **IBF_int** | **MDP_int** | 8,102 | 0.6238 (0.6218 - 0.6257) | 0.6583 (0.6556 - 0.6609) |
| **IBF_int** | **MDP_slope** | 8,102 | 0.6238 (0.6218 - 0.6257) | 0.4305 (0.4287 - 0.4322) |
| **IBF_int** | **BFT_int** | 8,431 | 0.6265 (0.6246 - 0.6283) | 0.7052 (0.7026 - 0.7077) |
| **IBF_int** | **BFT_slope** | 8,431 | 0.6265 (0.6246 - 0.6283) | 0.4446 (0.4427 - 0.4464) |
| **IBF_slope** | **OTW_int** | 41,380 | 0.4907 (0.4899 - 0.4914) | 0.6274 (0.6263 - 0.6283) |
| **IBF_slope** | **OTW_slope** | 41,380 | 0.4907 (0.4899 - 0.4914) | 0.5429 (0.5419 - 0.5438) |
| **IBF_slope** | **MDP_int** | 8,102 | 0.5407 (0.5387 - 0.5426) | 0.6583 (0.6556 - 0.6609) |
| **IBF_slope** | **MDP_slope** | 8,102 | 0.5407 (0.5387 - 0.5426) | 0.4305 (0.4287 - 0.4322) |
| **IBF_slope** | **BFT_int** | 8,431 | 0.5419 (0.5399 - 0.5438) | 0.7052 (0.7026 - 0.7077) |
| **IBF_slope** | **BFT_slope** | 8,431 | 0.5419 (0.5399 - 0.5438) | 0.4446 (0.4427 - 0.4464) |
| **OTW_int** | **MDP_int** | 17,218 | 0.6867 (0.6854 - 0.6879) | 0.6859 (0.6845 - 0.6873) |
| **OTW_int** | **MDP_slope** | 17,218 | 0.6867 (0.6854 - 0.6879) | 0.4188 (0.4176 - 0.4198) |
| **OTW_int** | **BFT_int** | 18,530 | 0.6902 (0.6890 - 0.6913) | 0.7350 (0.7337 - 0.7363) |
| **OTW_int** | **BFT_slope** | 18,530 | 0.6902 (0.6890 - 0.6913) | 0.4374 (0.4362 - 0.4384) |
| **OTW_slope** | **MDP_int** | 17,218 | 0.5998 (0.5985 - 0.6010) | 0.6859 (0.6845 - 0.6873) |
| **OTW_slope** | **MDP_slope** | 17,218 | 0.5998 (0.5985 - 0.6010) | 0.4188 (0.4176 - 0.4198) |
| **OTW_slope** | **BFT_int** | 18,530 | 0.6007 (0.5995 - 0.6018) | 0.7350 (0.7337 - 0.7363) |
| **OTW_slope** | **BFT_slope** | 18,530 | 0.6007 (0.5995 - 0.6018) | 0.4374 (0.4362 - 0.4384) |
| **MDP_int** | **BFT_int** | 12,231 | 0.6966 (0.6951 - 0.6981) | 0.7407 (0.7392 - 0.7421) |
| **MDP_int** | **BFT_slope** | 12,231 | 0.6966 (0.6951 - 0.6981) | 0.4429 (0.4415 - 0.4443) |
| **MDP_slope** | **BFT_int** | 12,231 | 0.4224 (0.4210 - 0.4236) | 0.7407 (0.7392 - 0.7421) |
| **MDP_slope** | **BFT_slope** | 12,231 | 0.4224 (0.4210 - 0.4236) | 0.4429 (0.4415 - 0.4443) |

^1^ OTW_30: off-test weight (Kg) considering an interval of 30 days; MDP_30: ultrasound muscle depth (mm) considering an interval of 30 days; BFT_30: ultrasound backfat thickness (mm) considering an interval of 30 days; TNB: total number of piglets born; NBA: number of piglets born alive; NBD: number of piglets born dead; IWE: interval between wean to estrus (days); IBF: interval between farrows (days); WN_wd: number of piglets weaned considering measurement until weaning date; WW_wd: weaning weight (Kg) considering measurement until weaning date.

^2^CI: confidence interval

**Table S7**. Spearman correlation between animals in reaction norms regressed on environmental variable and contemporary group effect, for the intercept and slope terms.

| **Spearman Correlation between GEBV** | | |
| --- | --- | --- |
| **Trait^1^** | **Spearman correlation** | |
|  | **Intercept** | **Slope** |
| **BFT** | 0.94 | -0.57 |
| **NBA** | 0.96 | 0.15 |
| **TNB** | 0.92 | 0.03 |
| **MDP** | 0.91 | -0.31 |
| **OTW** | 0.88 | 0.15 |
| **WN** | 0.71 | 0.148 |
| **WW** | 0.72 | -0.47 |

^1^ OTW: off-test weight (Kg); MDP: ultrasound muscle depth (mm); BFT: ultrasound backfat thickness (mm); TNB: total number of piglets born; NBA: number of piglets born alive; WN: number of piglets weaned; WW: weaning weight (Kg).
